# Supplementary material for: Analysis of the transcriptomic and metabolomic landscape of prostate cancer with different anatomical origins using snFLARE-seq and mxFRIZNGRND
Source: Nat Commun. 2026 Feb 7;17:2461. doi: 10.1038/s41467-026-69347-7 (PMC12992566; doi:10.1038/s41467-026-69347-7)
Supplement: Supplementary file 1 — Supplementary Information [file 41467_2026_69347_MOESM1_ESM.pdf]

Analysis of the transcriptomic and metabolomic landscape of prostate cancer with different anatomical origins using snFLARE-seq and mxFRIZNGRND

Dongyin He (何东印)[1,2, #], Haoran Hu (胡浩然)[3, #], Kai Xiao(肖楷)[2, #], Yuhang Zhang(张宇航)[2, #], Yongbing Cheng (程勇兵)[4, #], Shaozhuo Jiao (焦少灼)[5, #], Yimei Hao (郝一妹)[6, #], Yuanyuan Cai (蔡园园)[2], Ziqun Liu (刘子群)[2], Xinran Yan (颜欣然)[2], Qinsheng Chen (陈钦盛)[3], Xiyan Mu (穆希岩)[3], Qi Wang (王琪)[3], Shan Peng (彭姗)[7], Guoqin Sang (桑国芹)[5], Xiaoling Zhi (职小玲)[5], Yanxia Chang (常燕霞)[5], Qing Ye (叶青)[8], Yuyao Yang (杨羽尧)[8], Meixia Che (车美霞)[9], Shengsong Huang (黄盛松)[1, *],Hongqian Guo (郭宏骞)[4, *], Luonan Chen (陈洛南)[2,8,10, *], Huiru Tang (唐惠儒)[3, *], Xuefeng Qiu (邱雪峰)[4, *], Zhenfei Li (李振斐) [2,8 *]

[1] Department of Urology, Tongji Hospital, School of Medicine, Tongji University, Shanghai 200065, China

[2] Key Laboratory of Multi-Cell Systems, Shanghai Institute of Biochemistry and Cell Biology, Center for Excellence in Molecular Cell Science, Chinese Academy of Sciences; University of Chinese Academy of Sciences, 320 Yueyang Road, Shanghai 200031, China

[3] State Key Laboratory of Genetics and Development of Complex Phenotypes, School of Life Sciences, Human Phenome Institute, Zhangjiang Fudan International Innovation Center, Metabonomics and Systems Biology Laboratory at Shanghai International Center for Molecular Phenomics, Zhongshan Hospital, Fudan University, Shanghai 200032, China

[4] Department of Urology, Institute of Urology, Nanjing Drum Tower Hospital, Affiliated Hospital of Medical School, Nanjing University, Nanjing 210008, China;

[5] SeekGene BioSciences Co. Ltd, Beijing 102206, China

[6] Department of Laboratory Diagnostics, Changhai Hospital, Navy Military Medical University, Shanghai 200433, China

[7] Department of Pathology, Drum Tower Hospital, Medical School of Nanjing University, Institute of Urology, Nanjing University, Nanjing 210008, China

[8] Key Laboratory of Systems Health Science of Zhejiang Province, School of Life Science, Hangzhou Institute for Advanced Study, University of Chinese Academy of Sciences, Hangzhou 310024, China

[9] State Key Laboratory of Pharmaceutical Biotechnology, Chemistry and Biomedicine Innovation Center (ChemBIC), School of Life Sciences, Nanjing University, Nanjing 210023, China

[10] School of Mathematical Sciences and School of AI, Shanghai Jiao Tong University, Shanghai 200240, China

[#] These authors contribute equally to this work.

* **Correspondence:**

Zhenfei Li

320 Yueyang Road, Shanghai Institute of Biochemistry and Cell Biology, CAS Center for Excellence in Molecular Cell Science, Shanghai 200031, China

Phone: +86-21-54921339

Email: zhenfei.li@sibcb.ac.cn

Xuefeng Qiu

Email: xuefeng_qiu@nju.edu.cn

Huiru Tang

Email: huiru_tang@fudan.edu.cn

Luonan Chen

Email: lnchen@sjtu.edu.cn

Hongqian Guo

Email: dr.ghq@nju.edu.cn

Shengsong Huang

Email: hssfline@tongji.edu.cn

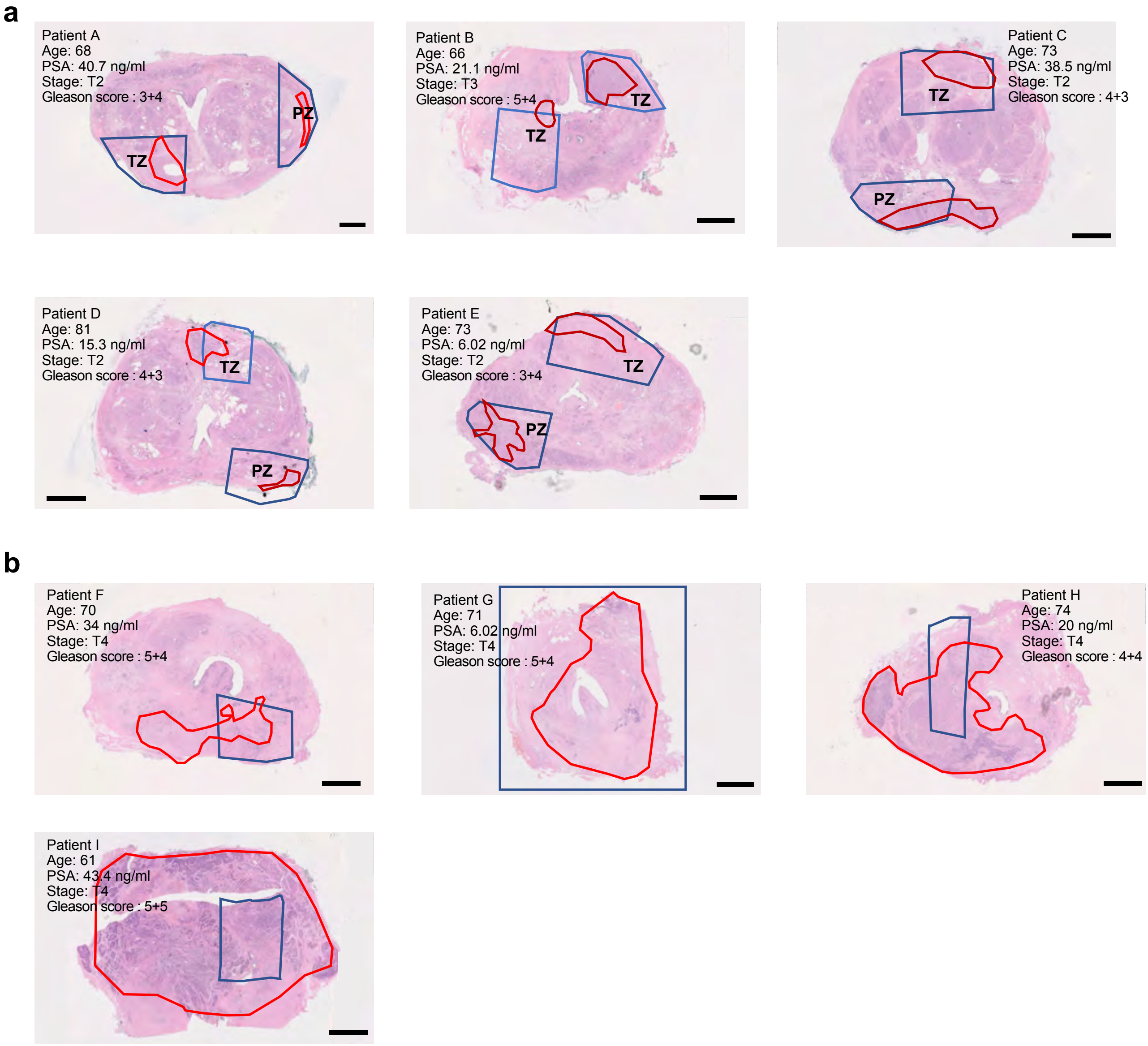
a
Patient A
Age: 68
PSA: 40.7 ng/ml
Stage: T2
Gleason score : 3+4
PZ
TZ
Patient B
Age: 66
PSA: 21.1 ng/ml
Stage: T3
Gleason score : 5+4
TZ
TZ
Patient C
Age: 73
PSA: 38.5 ng/ml
Stage: T2
Gleason score : 4+3
TZ
PZ
Patient D
Age: 81
PSA: 15.3 ng/ml
Stage: T2
Gleason score : 4+3
TZ
PZ
Patient E
Age: 73
PSA: 6.02 ng/ml
Stage: T2
Gleason score : 3+4
TZ
PZ
b
Patient F
Age: 70
PSA: 34 ng/ml
Stage: T4
Gleason score : 5+4
Patient G
Age: 71
PSA: 6.02 ng/ml
Stage: T4
Gleason score : 5+4
Patient H
Age: 74
PSA: 20 ng/ml
Stage: T4
Gleason score : 4+4
Patient I
Age: 61
PSA: 43.4 ng/ml
Stage: T4
Gleason score : 5+5

Supplementary Fig. 1| Pathological sampling for transcriptomic and metabolomic analysis. a, PZ (n = 4) and TZ (n = 6) samples from 5 patients. Paired PZ and TZ samples were collected from patient #A, #C, #D, and #E. Two TZ samples were collected from patient #B. **b,** PTM samples from patients receiving adjuvant therapy before prostatectomy (n = 4). Tumor areas span both PZ and TZ in these patients. Red line, tumor area; blue line, sample cutting area for further analysis.

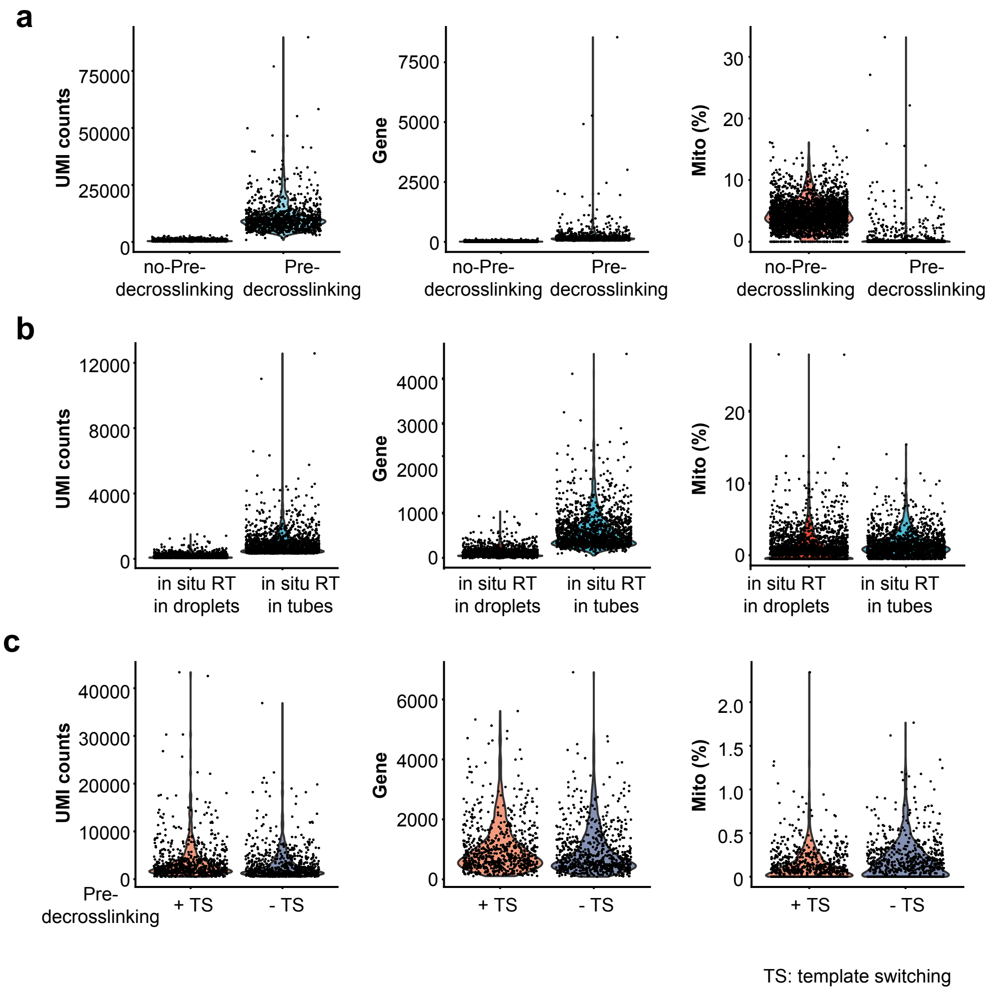
a
UMI counts
75000
50000
25000
0
no-Pre-decrosslinking
Pre-decrosslinking
Gene
7500
5000
2500
0
Mito (%)
30
20
10
0
b
UMI counts
12000
8000
4000
0
in situ RT in droplets
in situ RT in tubes
Gene
4000
3000
2000
1000
0
Mito (%)
20
10
0
c
UMI counts
40000
30000
20000
10000
0
Pre-decrosslinking
+ TS
- TS
Gene
6000
4000
2000
0
Mito (%)
2.0
1.5
1.0
0.5
0
TS: template switching

Supplementary Fig. 2| Overview and optimizations of snFLARE-seq. Pre-decrosslinking (**a**), in situ reverse transcription in tubes (**b**), and template switching (**c**) enhance gene detection and decrease mitochondrial contamination. RT, reverse transcription; TS, template switching. FFPE samples from human liver (n = 8) and kidney cancer (n = 2) were used for the optimization.

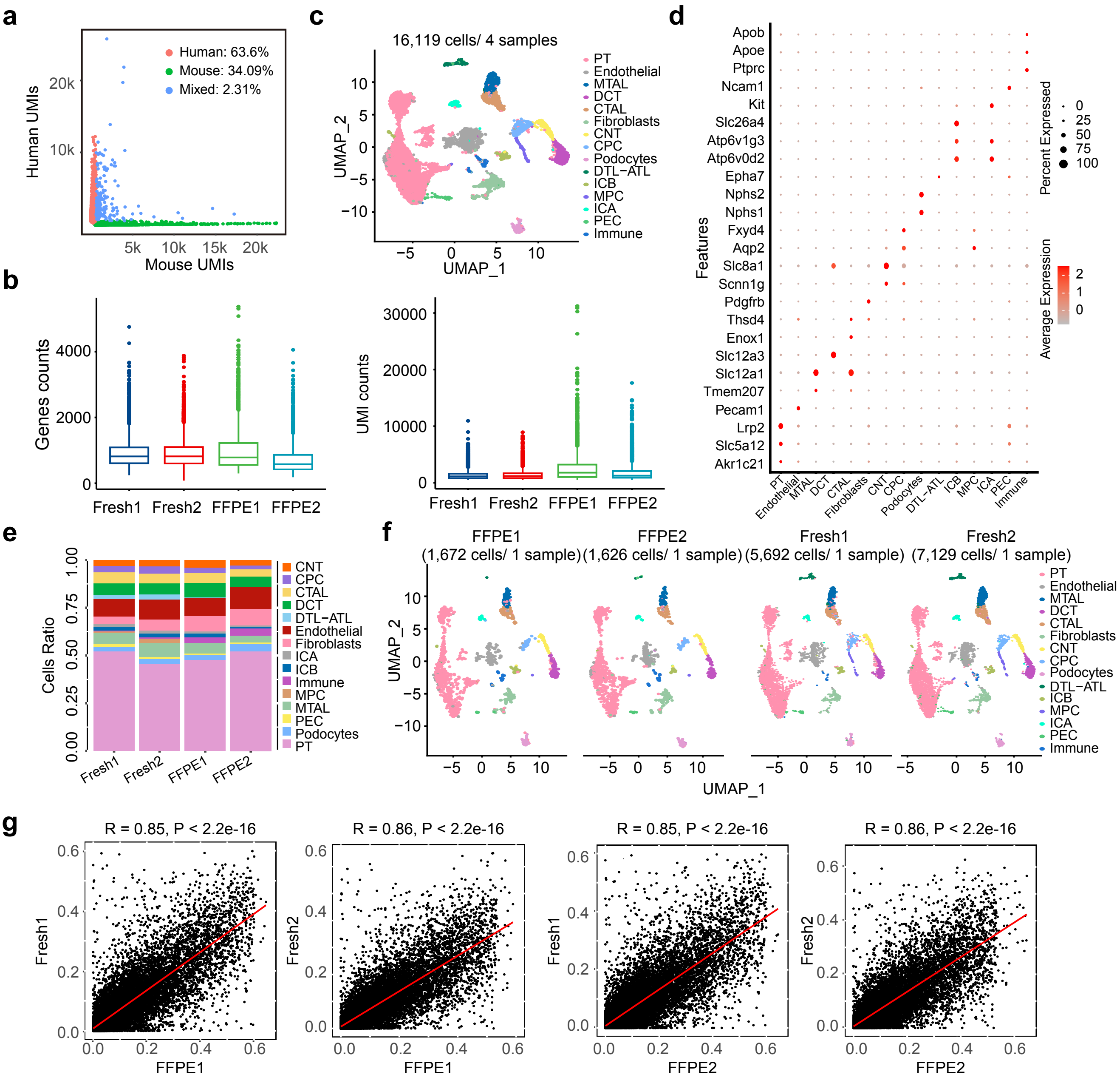
a
Human: 63.6%
Mouse: 34.09%
Mixed: 2.31%
Human UMIs
Mouse UMIs
c
16,119 cells/ 4 samples
UMAP_2
UMAP_1
PT
Endothelial
MTAL
DCT
CTAL
Fibroblasts
CNT
CPC
Podocytes
DTL−ATL
ICB
MPC
ICA
PEC
Immune
d
Apob
Apoe
Ptprc
Ncam1
Kit
Slc26a4
Atp6v1g3
Atp6v0d2
Epha7
Nphs2
Nphs1
Fxyd4
Aqp2
Slc8a1
Scnn1g
Pdgfrb
Thsd4
Enox1
Slc12a3
Slc12a1
Tmem207
Pecam1
Lrp2
Slc5a12
Akr1c21
Features
Percent Expressed
Average Expression
b
Genes counts
UMI counts
Fresh1
Fresh2
FFPE1
FFPE2
e
Cells Ratio
f
FFPE1 (1,672 cells/ 1 sample)
FFPE2 (1,626 cells/ 1 sample)
Fresh1 (5,692 cells/ 1 sample)
Fresh2 (7,129 cells/ 1 sample)
g
R = 0.85, P < 2.2e-16
R = 0.86, P < 2.2e-16
R = 0.85, P < 2.2e-16
R = 0.86, P < 2.2e-16

Supplementary Fig. 3| Validation of snFLARE. a, Doublete rate of snFLARE-seq with human-mouse mixed samples. FFPE human kidney cancer samples (1,672 cells from one sample) and FFPE mouse kidney cancer samples (1,626 cells from one sample) were used for the mixed species experiment. **b,** Gene counts detected in four samples (Fresh1 with 5,692 cells from one sample, Fresh2 with 7,129 cells from one sample, FFPE1 with 1,672 cells from one sample, and FFPE2 with 1,626 cells from one sample). Median, 25th percentile, and 75th percentile are shown in the box plot; whiskers extending to 1.5 X IQR (interquartile range). **c,** UMAP plot showing the identified 15 cell clusters from 16,119 cells from four samples. **d,** Dot plot of the average expression levels of top markers in each cluster. **e,** Distributions of different cell types in four samples. **f,** UMAP plot showing the distributions of different cell types in four samples (Fresh1 with 5,692 cells from one sample, Fresh2 with 7,129 cells from one sample, FFPE1 with 1,672 cells from one sample, and FFPE2 with 1,626 cells from one sample). **g,** Correlations of normalized gene expression profiles between fresh and FFPE samples. Pearson correlation analysis.

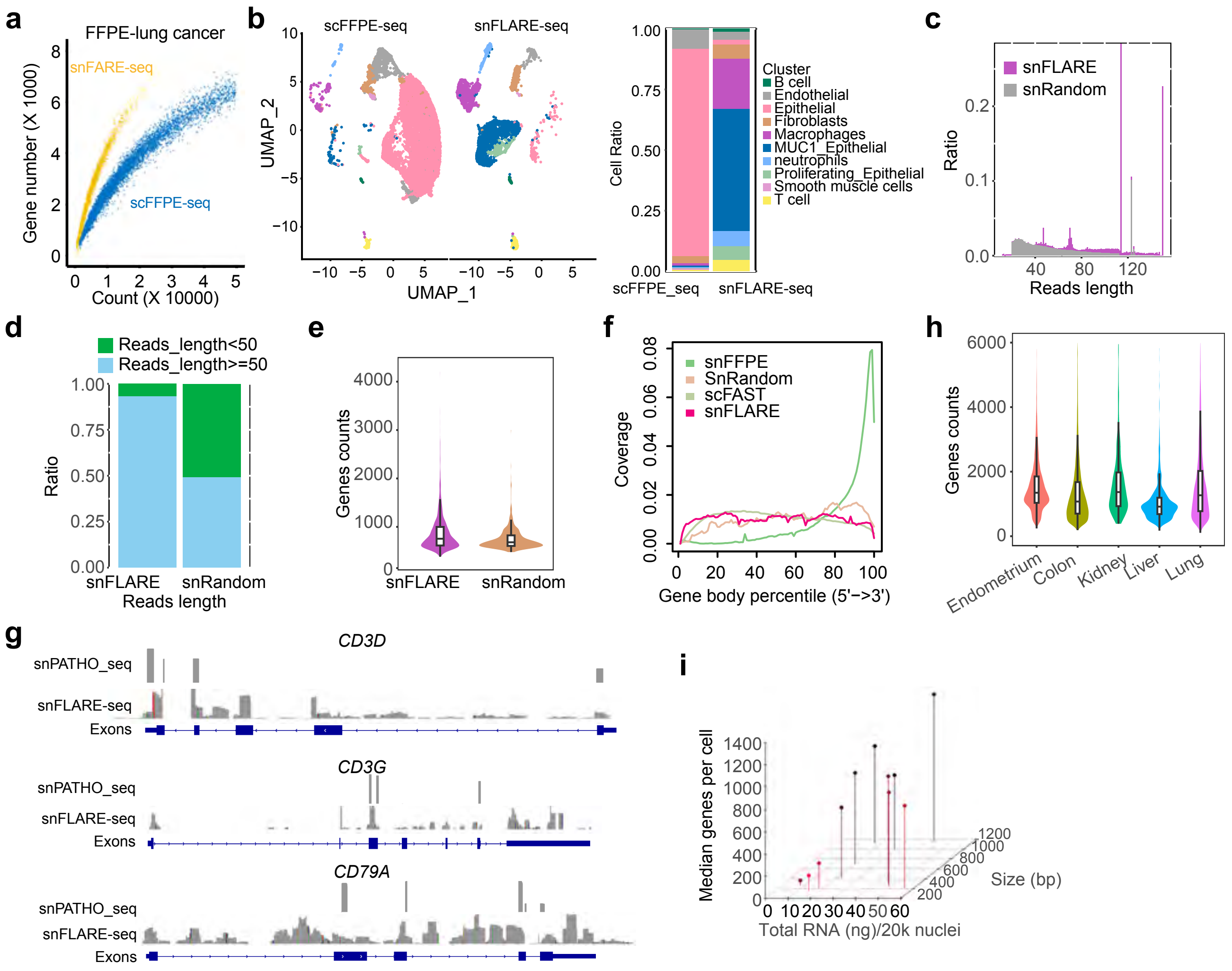
a
FFPE-lung cancer
snFARE-seq
scFFPE-seq
Gene number (X 1000)
Count (X 10000)
b
scFFPE-seq
snFLARE-seq
UMAP_2
UMAP_1
Cell Ratio
scFFPE_seq
snFLARE-seq
Cluster
B cell
Endothelial
Epithelial
Fibroblasts
Macrophages
MUC1_Epithelial
neutrophils
Proliferating_Epithelial
Smooth muscle cells
T cell
c
snFLARE
snRandom
Ratio
Reads length
d
Reads_length<50
Reads_length>=50
Ratio
snFLARE
snRandom
Reads length
e
Genes counts
snFLARE
snRandom
f
snFFPE
SnRandom
scFAST
snFLARE
Coverage
Gene body percentile (5'->3')
h
Genes counts
Endometrium
Colon
Kidney
Liver
Lung
g
CD3D
snPATHO_seq
snFLARE-seq
Exons
CD3G
snPATHO_seq
snFLARE-seq
Exons
CD79A
snPATHO_seq
snFLARE-seq
Exons
i
Median genes per cell
Total RNA (ng)/20k nuclei
Size (bp)

Supplementary Fig. 4| Comparison of snFLARE-seq with other FFPE snRNA-seq methods. **a**, Gene count comparison between scFFPE-seq (n = 1) and snFLARE-seq (n = 1) in lung cancer FFPE samples containing 7,019 cells. **b**, Cell type distribution profiles of scFFPE-seq (12,599 cells from one sample) versus snFLARE-seq (4962 cells from one sample). **c** and **d,** Distributions of read lengths from snFLARE-seq (n = 1) and snRandom-seq (n = 1). **e,** Gene counts from snFLARE-seq (n = 1) and snRandom-seq (n = 1). **f,** Read distributions across the gene body by different approaches (n = 4). **g,** Gene coverage breadth across genomic regions in snPATHO-seq and snFLARE-seq (n = 2). **h,** Genes detected in FFPE samples from different human tissue types (n = 11). Violin plots depict the kernel density of single-cell expression values. Median, 25th percentile, and 75th percentile are shown in the box plot; whiskers extending to 1.5 X IQR (interquartile range). **i,** Correlations of median genes per cell with RNA amount and RNA size.

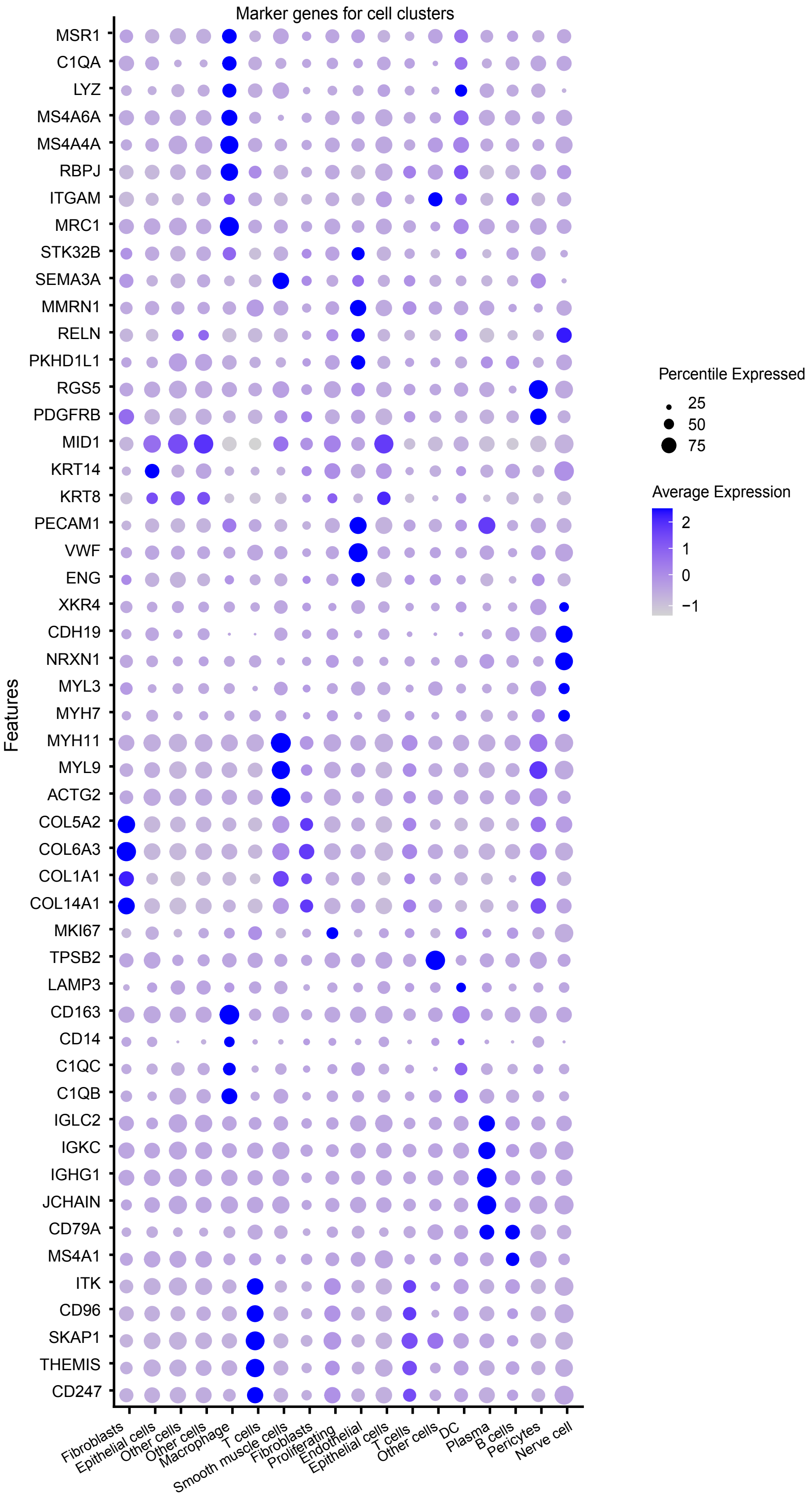

Marker genes for cell clusters
Features
MSR1
C1QA
LYZ
MS4A6A
MS4A4A
RBPJ
ITGAM
MRC1
STK32B
SEMA3A
MMRN1
RELN
PKHD1L1
RGS5
PDGFRB
MID1
KRT14
KRT8
PECAM1
VWF
ENG
XKR4
CDH19
NRXN1
MYL3
MYH7
MYH11
MYL9
ACTG2
COL5A2
COL6A3
COL1A1
COL14A1
MKI67
TPSB2
LAMP3
CD163
CD14
C1QC
C1QB
IGLC2
IGKC
IGHG1
JCHAIN
CD79A
MS4A1
ITK
CD96
SKAP1
THEMIS
CD247
Fibroblasts
Epithelial cells
Other cells
Other cells
Macrophage
T cells
Smooth muscle cells
Fibroblasts
Proliferating
Endothelial
Epithelial cells
T cells
Other cells
DC
Plasma
B cells
Pericytes
Nerve cell
Percentile Expressed
25
50
75
Average Expression
2
1
0
−1

Supplementary Fig. 5| Marker genes for cell types of snFLARE-seq results.

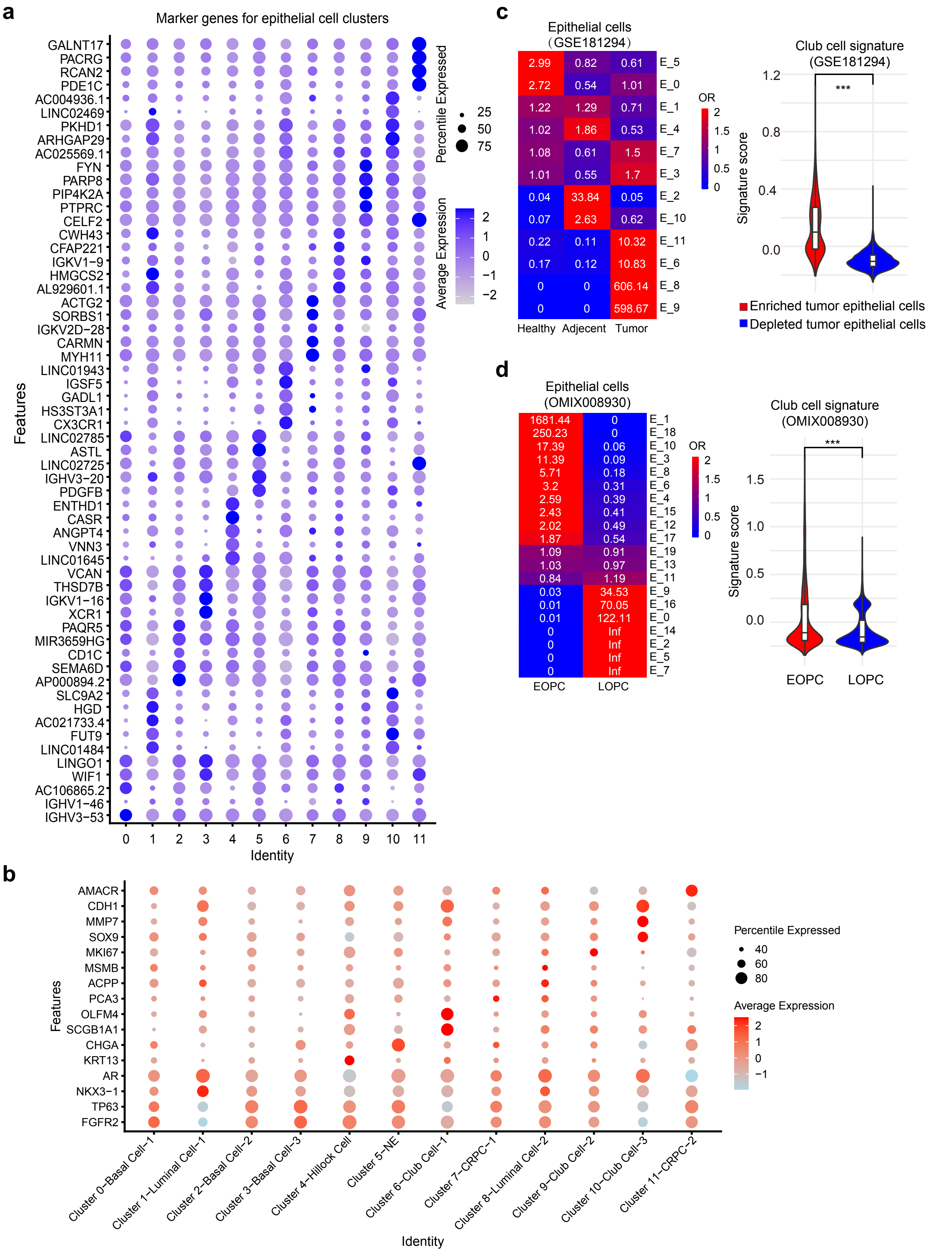
a
Marker genes for epithelial cell clusters
GALNT17
PACRG
RCAN2
PDE1C
AC004936.1
LINC02469
PKHD1
ARHGAP29
AC025569.1
FYN
PARP8
PIP4K2A
PTPRC
CELF2
CWH43
CFAP221
IGKV1−9
HMGCS2
AL929601.1
ACTG2
SORBS1
IGKV2D−28
CARMN
MYH11
LINC01943
IGSF5
GADL1
HS3ST3A1
CX3CR1
LINC02785
ASTL
LINC02725
IGHV3−20
PDGFB
ENTHD1
CASR
ANGPT4
VNN3
LINC01645
VCAN
THSD7B
IGKV1−16
XCR1
PAQR5
MIR3659HG
CD1C
SEMA6D
AP000894.2
SLC9A2
HGD
AC021733.4
FUT9
LINC01484
LINGO1
WIF1
AC106865.2
IGHV1−46
IGHV3−53
Features
0 1 2 3 4 5 6 7 8 9 10 11
Identity
Percentile Expressed
25
50
75
Average Expression
2
1
0
−1
−2
b
AMACR
CDH1
MMP7
SOX9
MKI67
MSMB
ACPP
PCA3
OLFM4
SCGB1A1
CHGA
KRT13
AR
NKX3−1
TP63
FGFR2
Features
Cluster 0−Basal Cell−1
Cluster 1−Luminal Cell−1
Cluster 2−Basal Cell−2
Cluster 3−Basal Cell−3
Cluster 4−Hillock Cell
Cluster 5−NE
Cluster 6−Club Cell−1
Cluster 7−CRPC−1
Cluster 8−Luminal Cell−2
Cluster 9−Club Cell−2
Cluster 10−Club Cell−3
Cluster 11−CRPC−2
Identity
Percentile Expressed
40
60
80
Average Expression
2
1
0
−1
c
Epithelial cells
（GSE181294）
2.99 0.82 0.61 E_5
2.72 0.54 1.01 E_0
1.22 1.29 0.71 E_1
1.02 1.86 0.53 E_4
1.08 0.61 1.5 E_7
1.01 0.55 1.7 E_3
0.04 33.84 0.05 E_2
0.07 2.63 0.62 E_10
0.22 0.11 10.32 E_11
0.17 0.12 10.83 E_6
0 0 606.14 E_8
0 0 598.67 E_9
Healthy Adjecent Tumor
OR
2
1.5
1
0.5
0
Club cell signature
(GSE181294)
***
Signature score
1.2
0.8
0.4
0.0
Enriched tumor epithelial cells
Depleted tumor epithelial cells
d
Epithelial cells
(OMIX008930)
1681.44 0 E_1
250.23 0 E_18
17.39 0.06 E_10
11.39 0.09 E_3
5.71 0.18 E_8
3.2 0.31 E_6
2.59 0.39 E_4
2.43 0.41 E_15
2.02 0.49 E_12
1.87 0.54 E_17
1.09 0.91 E_19
1.03 0.97 E_13
0.84 1.19 E_11
0.03 34.53 E_9
0.01 70.05 E_16
0.01 122.11 E_0
0 Inf E_14
0 Inf E_2
0 Inf E_5
0 Inf E_7
EOPC LOPC
OR
2
1.5
1
0.5
0
Club cell signature
(OMIX008930)
***
Signature score
1.5
1.0
0.5
0.0
EOPC
LOPC

Supplementary Fig. 6| Marker genes for epithelial clusters. a, Differentially expressed marker genes used for unsupervised clustering of epithelial cells. All of identified 16,415 epithelial cells from 14 samples were used for analysis. **b,** Clusters annotated using classic cell type markers. **c**, Club cells are enriched in tumor areas but not healthy prostate or noncancerous adjacent areas. 13,908 epithelial cells from GSE181294 dataset (1) were used for analysis. **d**, Club cells are enriched in early onset prostate cancer (EOPC) but not late onset prostate cancer (LOPC). 28,730 cells from OMIX008930 dataset (2) were used for analysis. Violin plots depict the kernel density of single-cell expression values. Median, 25th percentile, and 75th percentile are shown in the box plot; whiskers extending to 1.5 X IQR.

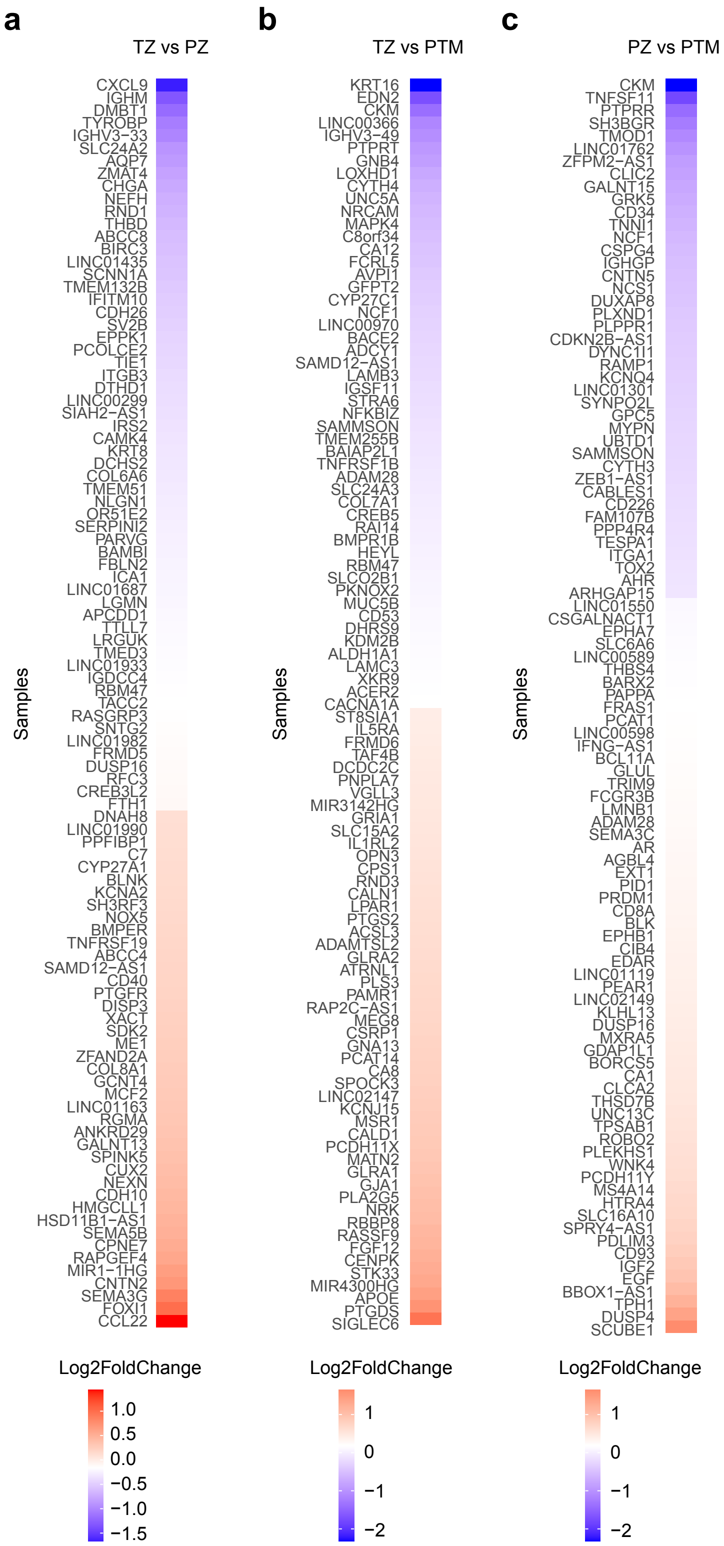
a
TZ vs PZ
Samples
CXCL9
IGHM
DMBT1
TYROBP
IGHV3−33
SLC24A2
AQP7
ZMAT4
CHGA
NEFH
RND1
THBD
ABCC8
BIRC3
LINC01435
SCNN1A
TMEM132B
IFITM10
CDH26
SV2B
EPPK1
PCOLCE2
TIE1
ITGB3
DTHD1
LINC00299
SIAH2−AS1
IRS2
CAMK4
KRT8
DCHS2
COL6A6
TMEM51
NLGN1
OR51E2
SERPINI2
PARVG
BAMBI
FBLN2
ICA1
LINC01687
LGMN
APCDD1
TTLL7
LRGUK
TMED3
LINC01933
IGDCC4
RBM47
TACC2
RASGRP3
SNTG2
LINC01982
FRMD5
DUSP16
RFC3
CREB3L2
FTH1
DNAH8
LINC01990
PPFIBP1
C7
CYP27A1
BLNK
KCNA2
SH3RF3
NOX5
BMPER
TNFRSF19
ABCC4
SAMD12−AS1
CD40
PTGFR
DISP3
XACT
SDK2
ME1
ZFAND2A
COL8A1
GCNT4
MCF2
LINC01163
RGMA
ANKRD29
GALNT13
SPINK5
CUX2
NEXN
CDH10
HMGCLL1
HSD11B1−AS1
SEMA5B
CPNE7
RAPGEF4
MIR1−1HG
CNTN2
SEMA3G
FOXI1
CCL22
Log2FoldChange
1.0
0.5
0.0
−0.5
−1.0
−1.5
b
TZ vs PTM
Samples
KRT16
EDN2
CKM
LINC00366
IGHV3−49
PTPRT
GNB4
LOXHD1
CYTH4
UNC5A
NRCAM
MAPK4
C8orf34
CA12
FCRL5
AVPI1
GFPT2
CYP27C1
NCF1
LINC00970
BACE2
ADCY1
SAMD12−AS1
LAMB3
IGSF11
STRA6
NFKBIZ
SAMMSON
TMEM255B
BAIAP2L1
TNFRSF1B
ADAM28
SLC24A3
COL7A1
CREB5
RAI14
BMPR1B
HEYL
RBM47
SLCO2B1
PKNOX2
MUC5B
CD53
DHRS9
KDM2B
ALDH1A1
LAMC3
XKR9
ACER2
CACNA1A
ST8SIA1
IL5RA
FRMD6
TAF4B
DCDC2C
PNPLA7
VGLL3
MIR3142HG
GRIA1
SLC15A2
IL1RL2
OPN3
CPS1
RND3
CALN1
LPAR1
PTGS2
ACSL3
ADAMTSL2
GLRA2
ATRNL1
PLS3
PAMR1
RAP2C−AS1
MEG8
CSRP1
GNA13
PCAT14
CA8
SPOCK3
LINC02147
KCNJ15
MSR1
CALD1
PCDH11X
MATN2
GLRA1
GJA1
PLA2G5
NRK
RBBP8
RASSF9
FGF12
CENPK
STK33
MIR4300HG
APOE
PTGDS
SIGLEC6
Log2FoldChange
1
0
−1
−2
c
PZ vs PTM
Samples
CKM
TNFSF11
PTPRR
SH3BGR
TMOD1
LINC01762
ZFPM2−AS1
CLIC2
GALNT15
GRK5
CD34
TNNI1
NCF1
CSPG4
IGHGP
CNTN5
NCS1
DUXAP8
PLXND1
PLPPR1
CDKN2B−AS1
DYNC1I1
RAMP1
KCNQ4
LINC01301
SYNPO2L
GPC5
MYPN
UBTD1
SAMMSON
CYTH3
ZEB1−AS1
CABLES1
CD226
FAM107B
PPP4R4
TESPA1
ITGA1
TOX2
AHR
ARHGAP15
LINC01550
CSGALNACT1
EPHA7
SLC6A6
LINC00589
THBS4
BARX2
PAPPA
FRAS1
PCAT1
LINC00598
IFNG−AS1
BCL11A
GLUL
TRIM9
FCGR3B
LMNB1
ADAM28
SEMA3C
AR
AGBL4
EXT1
PID1
PRDM1
CD8A
BLK
EPHB1
CIB4
EDAR
LINC01119
PEAR1
LINC02149
KLHL13
DUSP16
MXRA5
GDAP1L1
BORCS5
CA1
CLCA2
THSD7B
UNC13C
TPSAB1
ROBO2
PLEKHS1
WNK4
PCDH11Y
MS4A14
HTRA4
SLC16A10
SPRY4−AS1
PDLIM3
CD93
IGF2
EGF
BBOX1−AS1
TPH1
DUSP4
SCUBE1
Log2FoldChange
1
0
−1
−2

Supplementary Fig. 7| Differentially expressed genes in epithelial cells across three cohorts. a, Heatmap of sorted genes for GSEA between TZ (n = 6) and PZ (n = 4) samples. **b,** Heatmap of sorted genes for GSEA between TZ (n = 6) and PTM (n = 4) samples. **c,** Heatmap of sorted genes for GSEA between PZ (n = 4) and PTM (n = 4) samples.

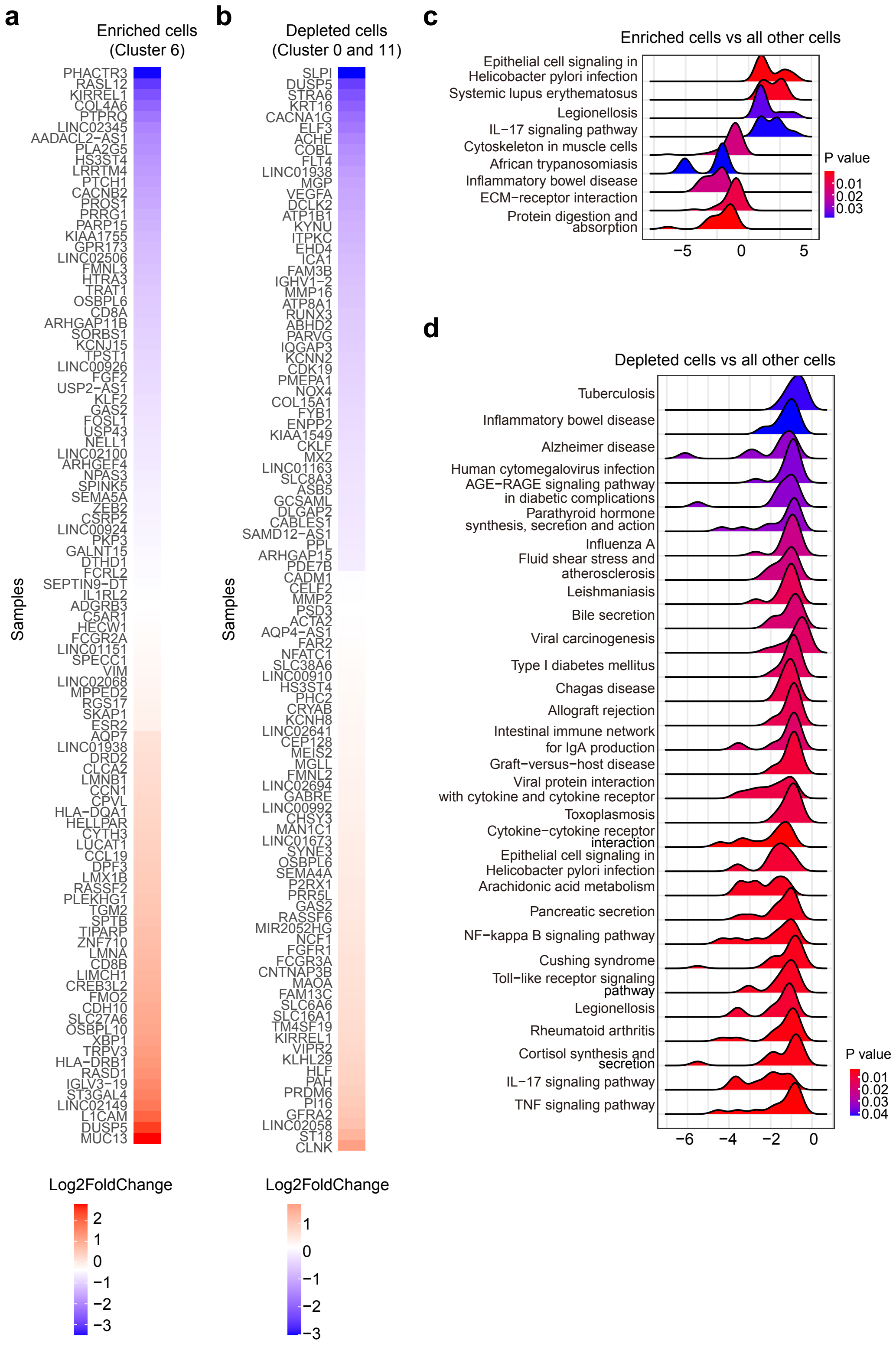
a
Enriched cells
(Cluster 6)
PHACTR3
RASL12
KIRREL1
COL4A6
PTPRQ
LINC02345
AADACL2−AS1
PLA2G5
HS3ST4
LRRTM4
PTCH1
CACNB2
PROS1
PRRG1
PARP15
KIAA1755
GPR173
LINC02506
FMNL3
HTRA3
TRAT1
OSBPL6
CD8A
ARHGAP11B
SORBS1
KCNJ15
TPST1
LINC00926
FGF2
USP2−AS1
KLF2
GAS2
FOSL1
USP43
NELL1
LINC02100
ARHGEF4
NPAS3
SPINK5
SEMA5A
ZEB2
CSRP2
LINC00924
PKP3
GALNT15
DTHD1
FCRL2
SEPTIN9−DT
IL1RL2
ADGRB3
C5AR1
HECW1
FCGR2A
LINC01151
SPECC1
VIM
LINC02068
MPPED2
RGS17
SKAP1
ESR2
AQP7
LINC01938
DRD2
CLCA2
LMNB1
CCN1
CPVL
HLA−DQA1
HELLPAR
CYTH3
LUCAT1
CCL19
DPF3
LMX1B
RASSF2
PLEKHG1
TGM2
SPTB
TIPARP
ZNF710
LMNA
CD8B
LIMCH1
CREB3L2
FMO2
CDH10
SLC27A6
OSBPL10
XBP1
TRPV3
HLA−DRB1
RASD1
IGLV3−19
ST3GAL4
LINC02149
L1CAM
DUSP5
MUC13
Samples
Log2FoldChange
2
1
0
−1
−2
−3
b
Depleted cells
(Cluster 0 and 11)
SLPI
DUSP5
STRA6
KRT16
CACNA1G
ELF3
ACHE
COBL
FLT4
LINC01938
MGP
VEGFA
DCLK2
ATP1B1
KYNU
ITPKC
EHD4
ICA1
FAM3B
IGHV1−2
MMP16
ATP8A1
RUNX3
ABHD2
PARVG
IQGAP3
KCNN2
CDK19
PMEPA1
NOX4
COL15A1
FYB1
ENPP2
KIAA1549
CKLF
MX2
LINC01163
SLC8A3
ASB5
GCSAML
DLGAP2
CABLES1
SAMD12−AS1
PPL
ARHGAP15
PDE7B
CADM1
CELF2
MMP2
PSD3
ACTA2
AQP4−AS1
FAR2
NFATC1
SLC38A6
LINC00910
HS3ST4
PHC2
CRYAB
KCNH8
LINC02641
CEP128
MEIS2
MGLL
FMNL2
LINC02694
GABRE
LINC00992
CHSY3
MAN1C1
LINC01673
SYNE3
OSBPL6
SEMA4A
P2RX1
PRR5L
GAS2
RASSF6
MIR2052HG
NCF1
FGFR1
FCGR3A
CNTNAP3B
MAOA
FAM13C
SLC6A6
SLC16A1
TM4SF19
KIRREL1
VIPR2
KLHL29
HLF
PAH
PRDM6
PI16
GFRA2
LINC02058
ST18
CLNK
Samples
Log2FoldChange
1
0
−1
−2
−3
c
Enriched cells vs all other cells
Epithelial cell signaling in
Helicobacter pylori infection
Systemic lupus erythematosus
Legionellosis
IL−17 signaling pathway
Cytoskeleton in muscle cells
African trypanosomiasis
Inflammatory bowel disease
ECM−receptor interaction
Protein digestion and
absorption
−5
0
5
P value
0.01
0.02
0.03
d
Depleted cells vs all other cells
Tuberculosis
Inflammatory bowel disease
Alzheimer disease
Human cytomegalovirus infection
AGE−RAGE signaling pathway
in diabetic complications
Parathyroid hormone
synthesis, secretion and action
Influenza A
Fluid shear stress and
atherosclerosis
Leishmaniasis
Bile secretion
Viral carcinogenesis
Type I diabetes mellitus
Chagas disease
Allograft rejection
Intestinal immune network
for IgA production
Graft−versus−host disease
Viral protein interaction
with cytokine and cytokine receptor
Toxoplasmosis
Cytokine−cytokine receptor
interaction
Epithelial cell signaling in
Helicobacter pylori infection
Arachidonic acid metabolism
Pancreatic secretion
NF−kappa B signaling pathway
Cushing syndrome
Toll−like receptor signaling
pathway
Legionellosis
Rheumatoid arthritis
Cortisol synthesis and
secretion
IL−17 signaling pathway
TNF signaling pathway
−6
−4
−2
0
P value
0.01
0.02
0.03
0.04

Supplementary Fig. 8| Differentially expressed genes in the enriched and depleted epithelial cell clusters. a, Heatmap of sorted genes for GSEA in enriched cells of PTM samples (n = 4). **b,** Heatmap of sorted genes for GSEA in depleted cells of PTM samples (n = 4). **c and d,** GSEA of the enriched and depleted clusters in PTM samples (n = 4). The statistical significance of enrichment was evaluated using GSEA, with a nominal P < 0.05 and a false discovery rate (FDR) < 0.25 considered statistically significant.

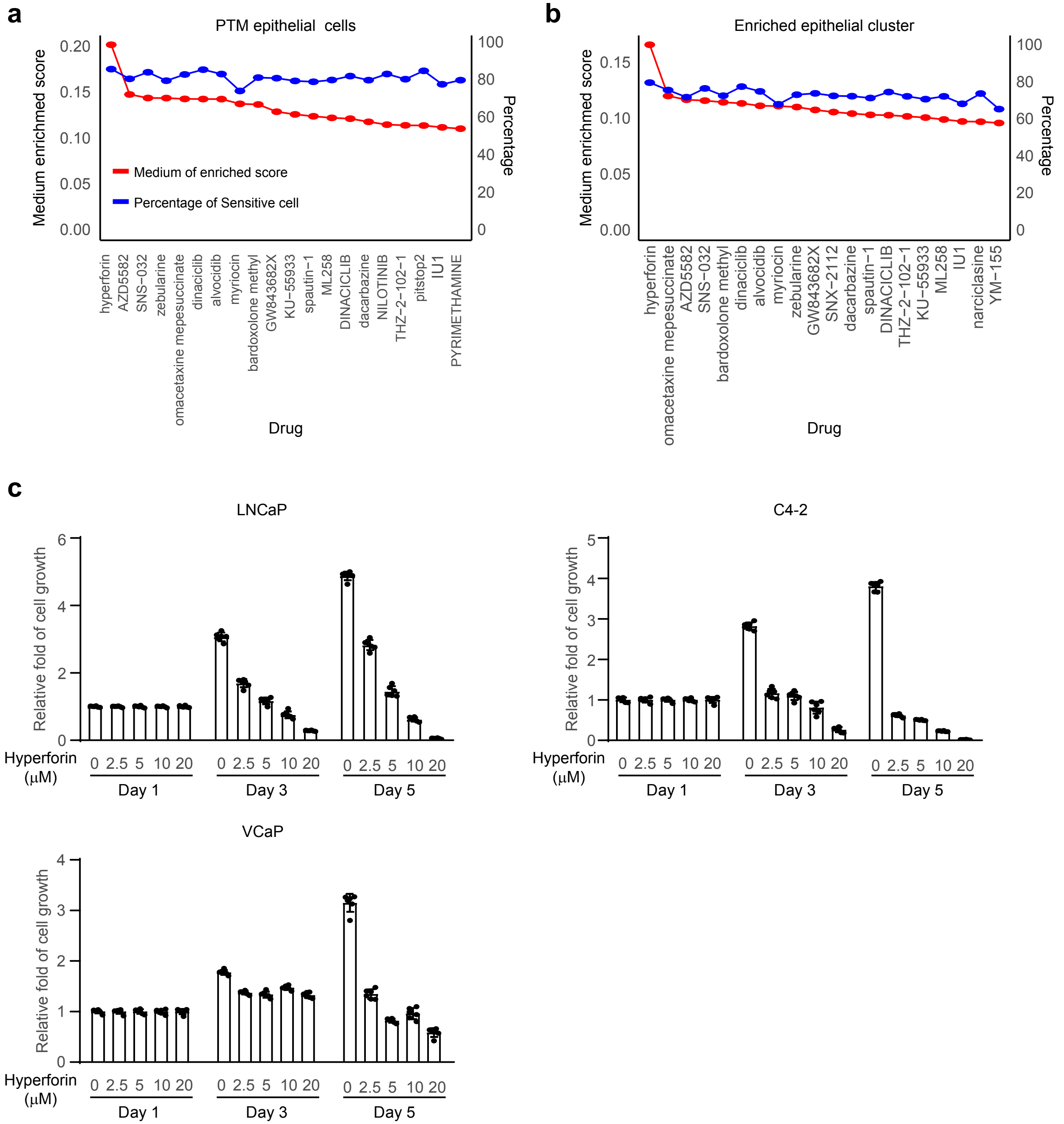

a
PTM epithelial cells
Medium enrichmed score
Percentage
Medium of enriched score
Percentage of Sensitive cell
hyperforin
AZD5582
SNS−032
zebularine
omacetaxine mepesuccinate
dinaciclib
alvocidib
myriocin
bardoxolone methyl
GW843682X
KU−55933
spautin−1
ML258
DINACICLIB
dacarbazine
NILOTINIB
THZ−2−102−1
pitstop2
IU1
PYRIMETHAMINE
Drug
b
Enriched epithelial cluster
Medium enrichmed score
Percentage
hyperforin
omacetaxine mepesuccinate
AZD5582
SNS−032
bardoxolone methyl
dinaciclib
alvocidib
myriocin
zebularine
GW843682X
SNX−2112
dacarbazine
spautin−1
DINACICLIB
THZ−2−102−1
KU−55933
ML258
IU1
narciclasine
YM−155
Drug
c
LNCaP
C4-2
VCaP
Relative fold of cell growth
Hyperforin (μM)
0 2.5 5 10 20
Day 1
Day 3
Day 5

Supplementary Fig. 9| Potential drugs for prostate cancer based on transcriptomic features of epithelial cells in PTM samples. a and b, Transcriptomic features of epithelial cells (a) or enriched clusters (b) in PTM samples were used for DREEP (Drug Response Estimation from single-cell Expression Profiles) analysis. Left Y-axis, the median enrichment score; right Y-axis, percentage of sensitive cells. **c,** Effect of hyperforin on cell growth in LNCaP, C4-2, and VCaP cells. Results were shown as mean ± SD; n = 6 independent replicates. Source data are provided as a Source Data file.

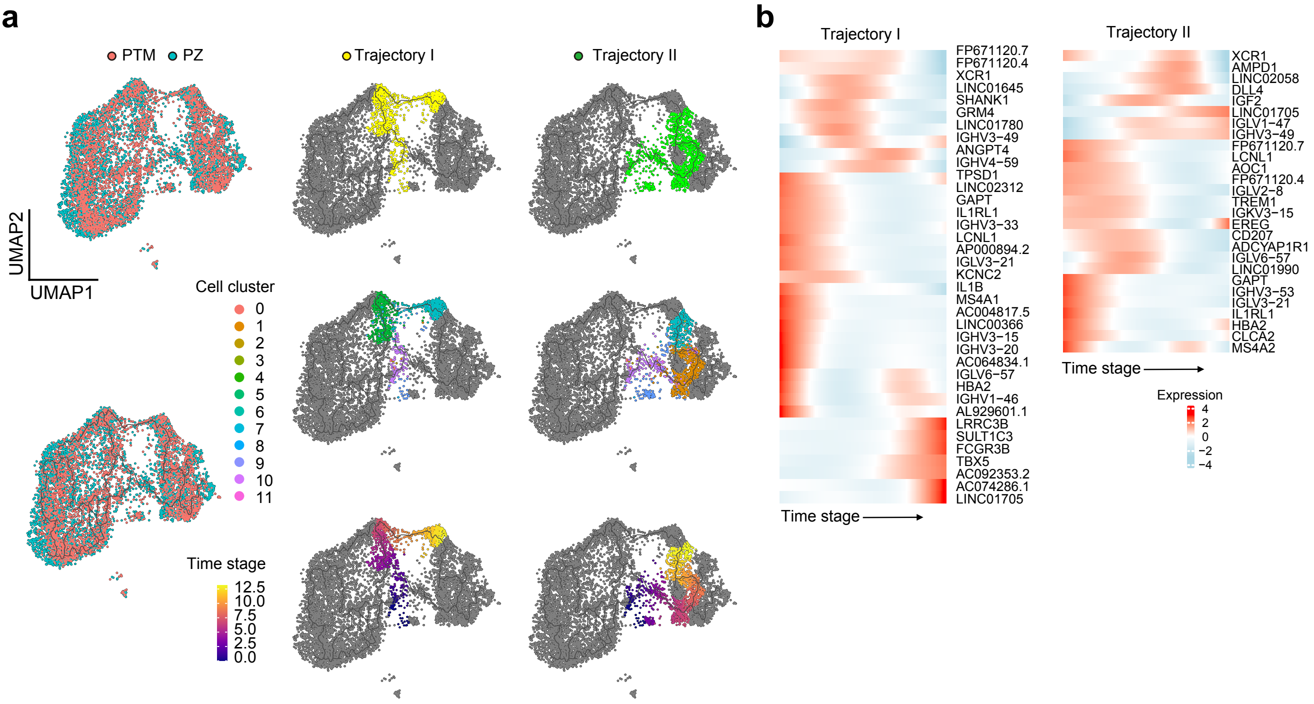
a
PTM
PZ
Trajectory I
Trajectory II
UMAP2
UMAP1
Cell cluster
0
1
2
3
4
5
6
7
8
9
10
11
Time stage
12.5
10.0
7.5
5.0
2.5
0.0
b
Trajectory I
FP671120.7
FP671120.4
XCR1
LINC01645
SHANK1
GRM4
LINC01780
IGHV3−49
ANGPT4
IGHV4−59
TPSD1
LINC02312
GAPT
IL1RL1
IGHV3−33
LCNL1
AP000894.2
IGLV3−21
KCNC2
IL1B
MS4A1
AC004817.5
LINC00366
IGHV3−15
IGHV3−20
AC064834.1
IGLV6−57
HBA2
IGHV1−46
AL929601.1
LRRC3B
SULT1C3
FCGR3B
TBX5
AC092353.2
AC074286.1
LINC01705
Time stage
Trajectory II
XCR1
AMPD1
LINC02058
DLL4
IGF2
LINC01705
IGLV1−47
IGHV3−49
FP671120.7
LCNL1
AOC1
FP671120.4
IGLV2−8
TREM1
IGKV3−15
EREG
CD207
ADCYAP1R1
IGLV6−57
LINC01990
GAPT
IGHV3−53
IGLV3−21
IL1RL1
HBA2
CLCA2
MS4A2
Time stage
Expression
4
2
0
−2
−4

Supplementary Fig. 10| Dynamic network biomarkers (DNB) analysis on cell evolution. a, Trajectory plots with epithelial cells from PZ (n = 4) and PTM (n = 4) samples. Cluster 9 was the starting point and cluster 6 as the endpoint. Two trajectories, related clusters, and pseudotime changes were displayed. **b,** Genes with significant expression differences over pseudotime in two trajectories.

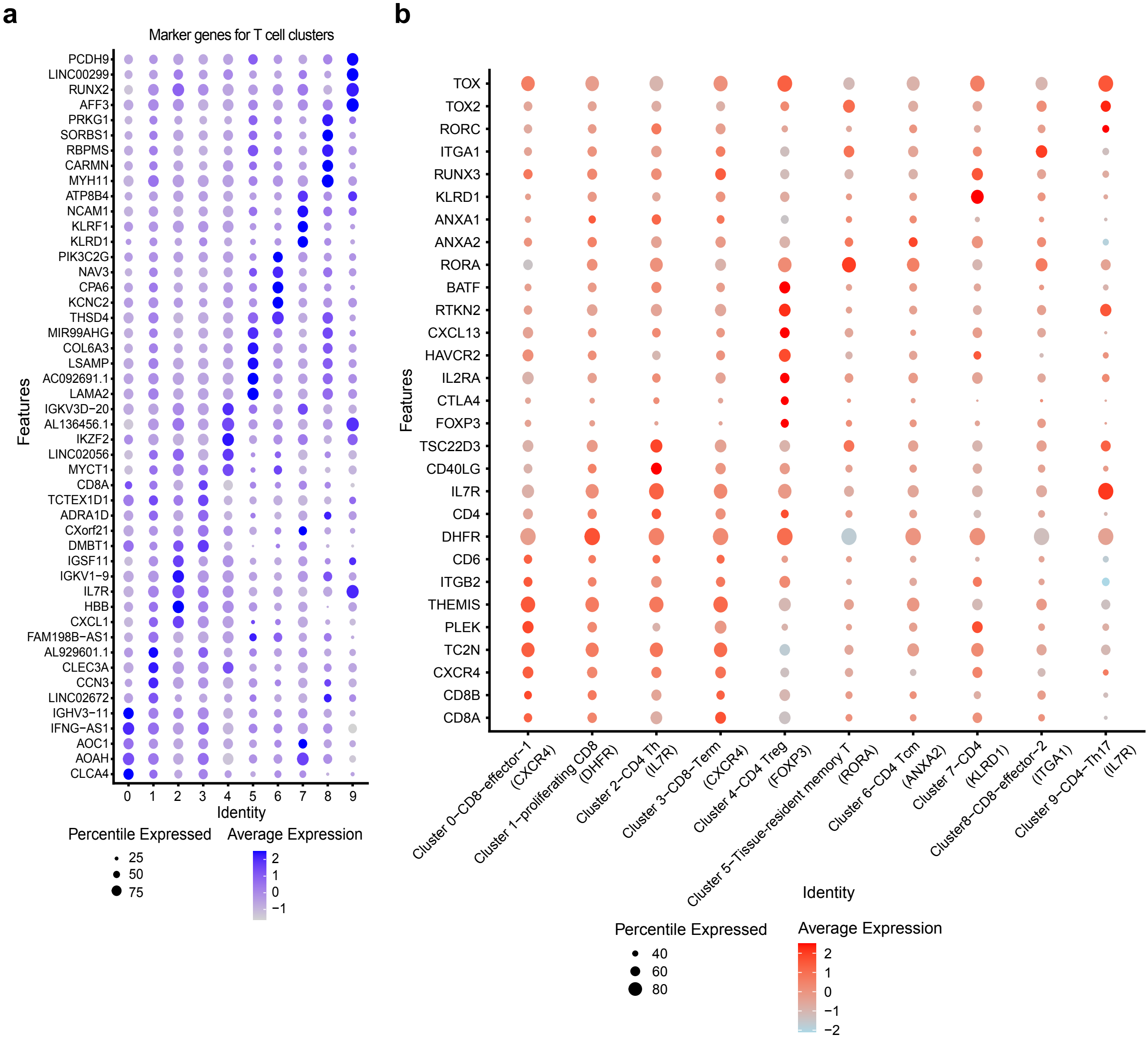
a
Marker genes for T cell clusters
PCDH9
LINC00299
RUNX2
AFF3
PRKG1
SORBS1
RBPMS
CARMN
MYH11
ATP8B4
NCAM1
KLRF1
KLRD1
PIK3C2G
NAV3
CPA6
KCNC2
THSD4
MIR99AHG
COL6A3
LSAMP
AC092691.1
LAMA2
IGKV3D−20
AL136456.1
IKZF2
LINC02056
MYCT1
CD8A
TCTEX1D1
ADRA1D
CXorf21
DMBT1
IGSF11
IGKV1−9
IL7R
HBB
CXCL1
FAM198B−AS1
AL929601.1
CLEC3A
CCN3
LINC02672
IGHV3−11
IFNG−AS1
AOC1
AOAH
CLCA4
Features
0 1 2 3 4 5 6 7 8 9
Identity
Percentile Expressed
25
50
75
Average Expression
2
1
0
−1
b
TOX
TOX2
RORC
ITGA1
RUNX3
KLRD1
ANXA1
ANXA2
RORA
BATF
RTKN2
CXCL13
HAVCR2
IL2RA
CTLA4
FOXP3
TSC22D3
CD40LG
IL7R
CD4
DHFR
CD6
ITGB2
THEMIS
PLEK
TC2N
CXCR4
CD8B
CD8A
Features
Cluster 0−CD8−effector−1 (CXCR4)
Cluster 1−proliferating CD8 (DHFR)
Cluster 2−CD4 Th (IL7R)
Cluster 3−CD8−Term (CXCR4)
Cluster 4−CD4 Treg (FOXP3)
Cluster 5−Tissue-resident memory T (RORA)
Cluster 6−CD4 Tcm (ANXA2)
Cluster 7−CD4 (KLRD1)
Cluster8−CD8−effector−2 (ITGA1)
Cluster 9−CD4−Th17 (IL7R)
Identity
Percentile Expressed
40
60
80
Average Expression
2
1
0
−1
−2

Supplementary Fig. 11| Marker genes for T cell clusters. a, Differentially expressed marker genes used for unsupervised clustering of epithelial cells. **b,** Clusters annotated using classic cell type markers.

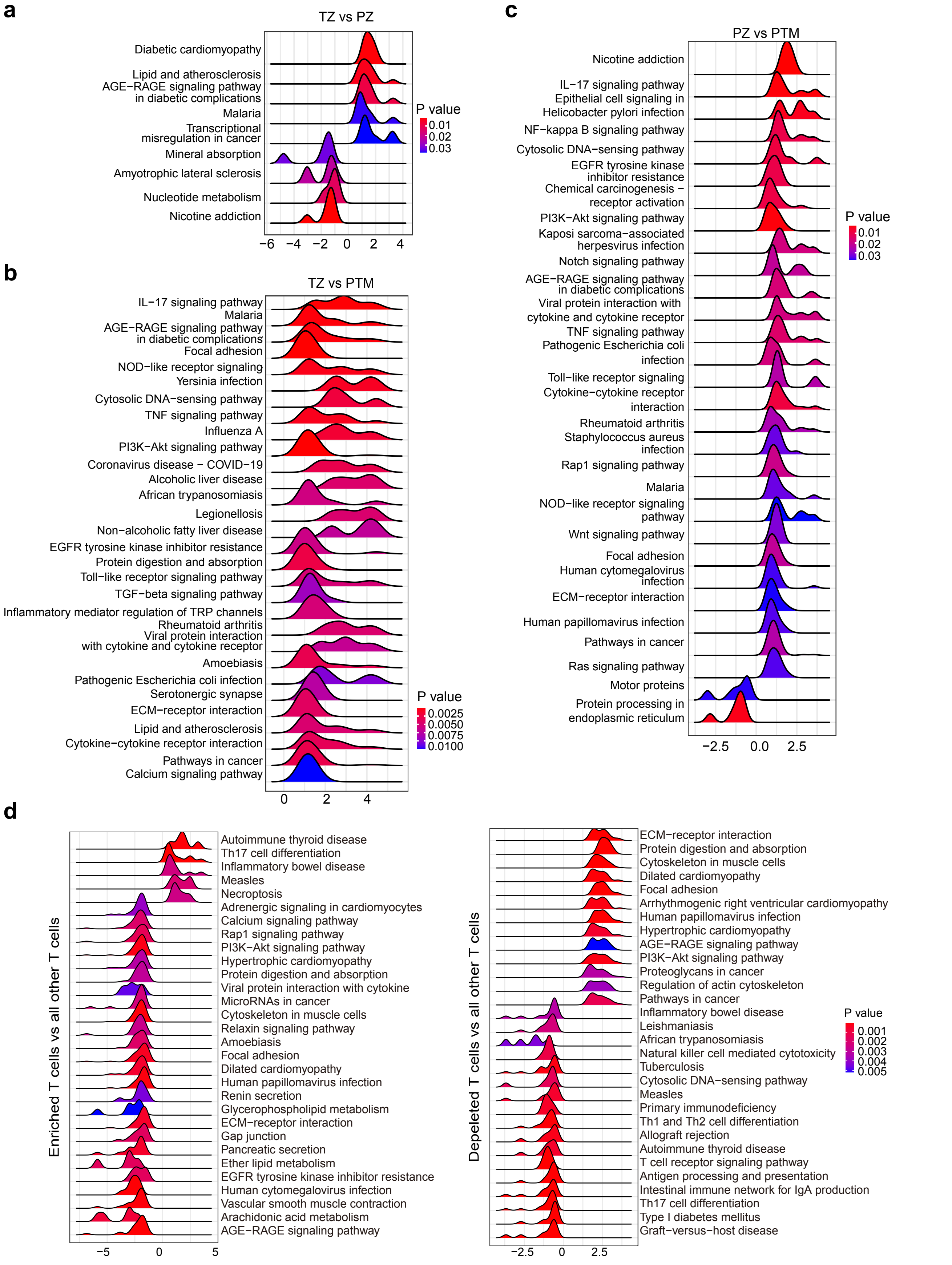
a
TZ vs PZ
Diabetic cardiomyopathy
Lipid and atherosclerosis
AGE−RAGE signaling pathway in diabetic complications
Malaria
Transcriptional misregulation in cancer
Mineral absorption
Amyotrophic lateral sclerosis
Nucleotide metabolism
Nicotine addiction
−6 −4 −2 0 2 4
P value
0.01
0.02
0.03
b
TZ vs PTM
IL−17 signaling pathway
Malaria
AGE−RAGE signaling pathway in diabetic complications
Focal adhesion
NOD−like receptor signaling
Yersinia infection
Cytosolic DNA−sensing pathway
TNF signaling pathway
Influenza A
PI3K−Akt signaling pathway
Coronavirus disease − COVID−19
Alcoholic liver disease
African trypanosomiasis
Legionellosis
Non−alcoholic fatty liver disease
EGFR tyrosine kinase inhibitor resistance
Protein digestion and absorption
Toll−like receptor signaling pathway
TGF−beta signaling pathway
Inflammatory mediator regulation of TRP channels
Rheumatoid arthritis
Viral protein interaction with cytokine and cytokine receptor
Amoebiasis
Pathogenic Escherichia coli infection
Serotonergic synapse
ECM−receptor interaction
Lipid and atherosclerosis
Cytokine−cytokine receptor interaction
Pathways in cancer
Calcium signaling pathway
0 2 4
P value
0.0025
0.0050
0.0075
0.0100
c
PZ vs PTM
Nicotine addiction
IL−17 signaling pathway
Epithelial cell signaling in Helicobacter pylori infection
NF−kappa B signaling pathway
Cytosolic DNA−sensing pathway
EGFR tyrosine kinase inhibitor resistance
Chemical carcinogenesis − receptor activation
PI3K−Akt signaling pathway
Kaposi sarcoma−associated herpesvirus infection
Notch signaling pathway
AGE−RAGE signaling pathway in diabetic complications
Viral protein interaction with cytokine and cytokine receptor
TNF signaling pathway
Pathogenic Escherichia coli infection
Toll−like receptor signaling
Cytokine−cytokine receptor interaction
Rheumatoid arthritis
Staphylococcus aureus infection
Rap1 signaling pathway
Malaria
NOD−like receptor signaling pathway
Wnt signaling pathway
Focal adhesion
Human cytomegalovirus infection
ECM−receptor interaction
Human papillomavirus infection
Pathways in cancer
Ras signaling pathway
Motor proteins
Protein processing in endoplasmic reticulum
−2.5 0.0 2.5
P value
0.01
0.02
0.03
d
Enriched T cells vs all other T cells
Autoimmune thyroid disease
Th17 cell differentiation
Inflammatory bowel disease
Measles
Necroptosis
Adrenergic signaling in cardiomyocytes
Calcium signaling pathway
Rap1 signaling pathway
PI3K−Akt signaling pathway
Hypertrophic cardiomyopathy
Protein digestion and absorption
Viral protein interaction with cytokine
MicroRNAs in cancer
Cytoskeleton in muscle cells
Relaxin signaling pathway
Amoebiasis
Focal adhesion
Dilated cardiomyopathy
Human papillomavirus infection
Renin secretion
Glycerophospholipid metabolism
ECM−receptor interaction
Gap junction
Pancreatic secretion
Ether lipid metabolism
EGFR tyrosine kinase inhibitor resistance
Human cytomegalovirus infection
Vascular smooth muscle contraction
Arachidonic acid metabolism
AGE−RAGE signaling pathway
−5 0 5
Depeleted T cells vs all other T cells
ECM−receptor interaction
Protein digestion and absorption
Cytoskeleton in muscle cells
Dilated cardiomyopathy
Focal adhesion
Arrhythmogenic right ventricular cardiomyopathy
Human papillomavirus infection
Hypertrophic cardiomyopathy
AGE−RAGE signaling pathway
PI3K−Akt signaling pathway
Proteoglycans in cancer
Regulation of actin cytoskeleton
Pathways in cancer
Inflammatory bowel disease
Leishmaniasis
African trypanosomiasis
Natural killer cell mediated cytotoxicity
Tuberculosis
Cytosolic DNA−sensing pathway
Measles
Primary immunodeficiency
Th1 and Th2 cell differentiation
Allograft rejection
Autoimmune thyroid disease
T cell receptor signaling pathway
Antigen processing and presentation
Intestinal immune network for IgA production
Th17 cell differentiation
Type I diabetes mellitus
Graft−versus−host disease
−2.5 0 2.5
P value
0.001
0.002
0.003
0.004
0.005

Supplementary Fig. 12| GSEA of T cells from different cohorts. a, Differentially enriched pathways between TZ (n = 6) and PZ (n = 4) samples. **b,** Differentially enriched pathways between TZ (n = 6) and PTM (n = 4) samples. **c,** Differentially enriched pathways between PZ (n = 4) and PTM (n = 4) samples. **d,** GSEA of the enriched and depleted T cell clusters in PTM samples (n = 4). Statistical significance was determined using the GSEA method, with a nominal $P < 0.05$ and a false discovery rate (FDR) < 0.25 considered statistically significant.

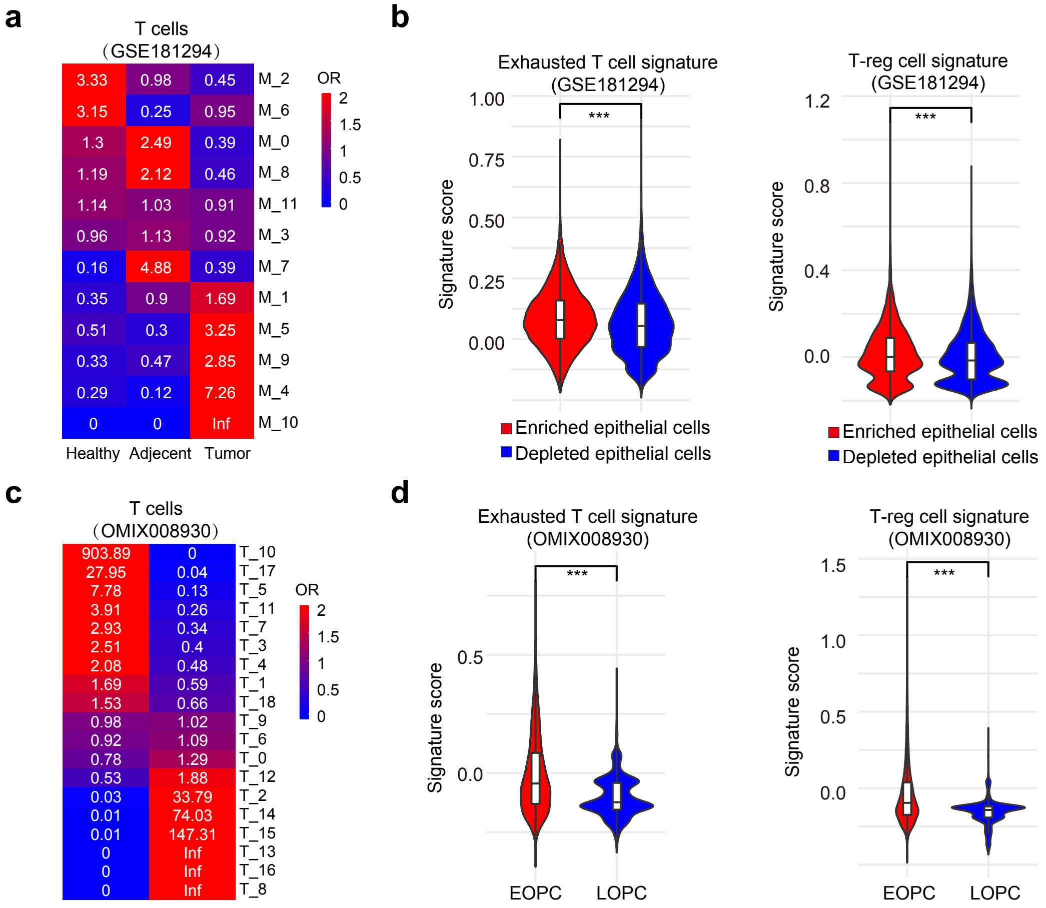
a
T cells
(GSE181294)
OR
Healthy Adjecent Tumor
M_2 M_6 M_0 M_8 M_11 M_3 M_7 M_1 M_5 M_9 M_4 M_10
3.33 0.98 0.45
3.15 0.25 0.95
1.3 2.49 0.39
1.19 2.12 0.46
1.14 1.03 0.91
0.96 1.13 0.92
0.16 4.88 0.39
0.35 0.9 1.69
0.51 0.3 3.25
0.33 0.47 2.85
0.29 0.12 7.26
0 0 Inf
b
Exhausted T cell signature
(GSE181294)
T-reg cell signature
(GSE181294)
Signature score
***
Enriched epithelial cells
Depleted epithelial cells
c
T cells
(OMIX008930)
T_10 T_17 T_5 T_11 T_7 T_3 T_4 T_1 T_18 T_9 T_6 T_0 T_12 T_2 T_14 T_15 T_13 T_16 T_8
903.89 0
27.95 0.04
7.78 0.13
3.91 0.26
2.93 0.34
2.51 0.4
2.08 0.48
1.69 0.59
1.53 0.66
0.98 1.02
0.92 1.09
0.78 1.29
0.53 1.88
0.03 33.79
0.01 74.03
0.01 147.31
0 Inf
0 Inf
0 Inf
d
Exhausted T cell signature
(OMIX008930)
T-reg cell signature
(OMIX008930)
Signature score
***
EOPC
LOPC

Supplementary Fig. 13| Validation signatures of tumor immune microenvironment in external databases. a and b, Exhausted T cells and Treg cells are enriched in tumor areas but not healthy prostate or noncancerous adjacent areas. 54,107 cells from GSE181294 dataset (1) were used for analysis. Cluster–tissue enrichment was quantified by odds ratios (ORs) derived from Fisher's exact test, with P values adjusted using the Benjamini–Hochberg method. Enrichment or depletion was considered significant when OR > 1.5 or OR < 0.5 and adjusted $P < 1 \times 10^{-10}$. Violin plots depict the kernel density of single-cell expression values. Median, 25th percentile, and 75th percentile are shown in the box plot; whiskers extending to 1.5 X IQR. **c and d,** Exhausted T cells and Treg cells are enriched in early onset prostate cancer (EOPC) but not late onset prostate cancer (LOPC). 29,804 cells from OMIX008930 dataset (2) were used for analysis. Differences between groups were assessed using the two-sided Wilcoxon rank-sum test, and P values were adjusted by the Benjamini–Hochberg method. Statistical significance was defined as adjusted P < 0.05, with significance levels denoted as * P < 0.05; ** P < 0.01; *** P < 0.001** in the violin plots.

a

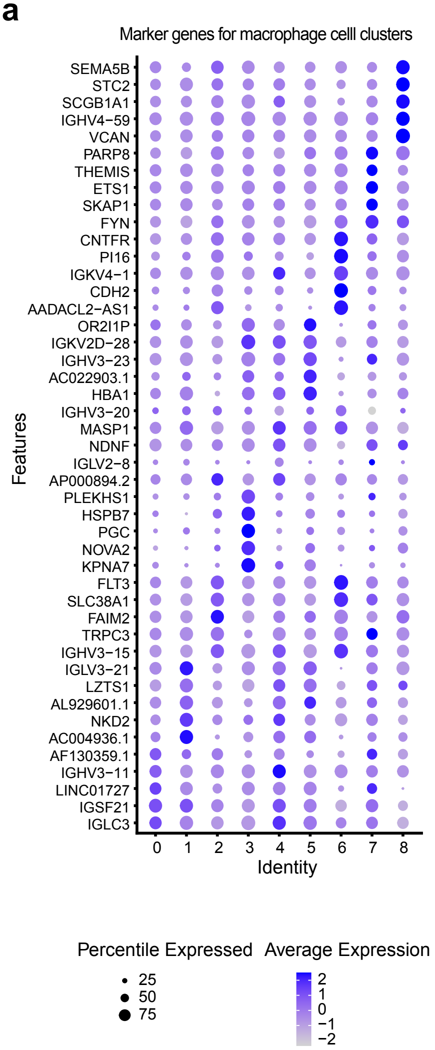
Marker genes for macrophage celll clusters
Features
SEMA5B
STC2
SCGB1A1
IGHV4−59
VCAN
PARP8
THEMIS
ETS1
SKAP1
FYN
CNTFR
PI16
IGKV4−1
CDH2
AADACL2−AS1
OR2I1P
IGKV2D−28
IGHV3−23
AC022903.1
HBA1
IGHV3−20
MASP1
NDNF
IGLV2−8
AP000894.2
PLEKHS1
HSPB7
PGC
NOVA2
KPNA7
FLT3
SLC38A1
FAIM2
TRPC3
IGHV3−15
IGLV3−21
LZTS1
AL929601.1
NKD2
AC004936.1
AF130359.1
IGHV3−11
LINC01727
IGSF21
IGLC3
0 1 2 3 4 5 6 7 8
Identity
Percentile Expressed
25
50
75
Average Expression
2
1
0
−1
−2

b

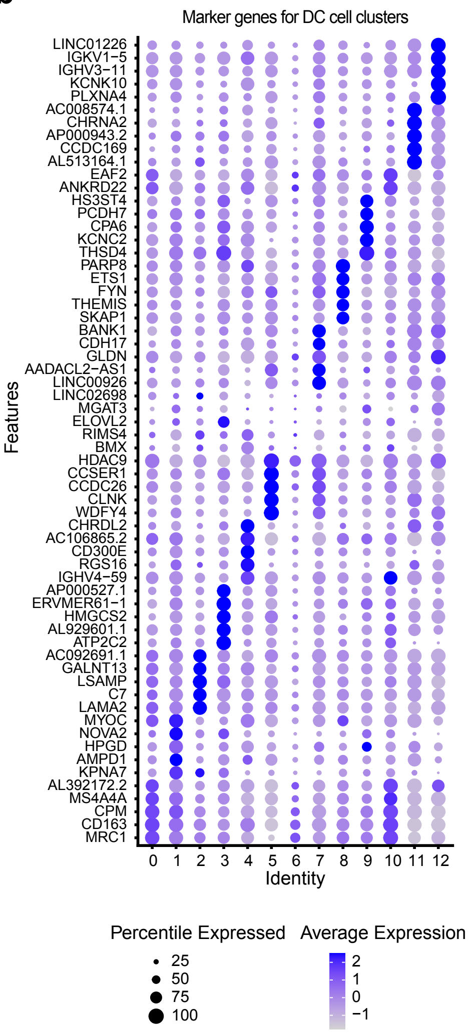
Marker genes for DC cell clusters
Features
LINC01226
IGKV1−5
IGHV3−11
KCNK10
PLXNA4
AC008574.1
CHRNA2
AP000943.2
CCDC169
AL513164.1
EAF2
ANKRD22
HS3ST4
PCDH7
CPA6
KCNC2
THSD4
PARP8
ETS1
FYN
THEMIS
SKAP1
BANK1
CDH17
GLDN
AADACL2−AS1
LINC00926
LINC02698
MGAT3
ELOVL2
RIMS4
BMX
HDAC9
CCSER1
CCDC26
CLNK
WDFY4
CHRDL2
AC106865.2
CD300E
RGS16
IGHV4−59
AP000527.1
ERVMER61−1
HMGCS2
AL929601.1
ATP2C2
AC092691.1
GALNT13
LSAMP
C7
LAMA2
MYOC
NOVA2
HPGD
AMPD1
KPNA7
AL392172.2
MS4A4A
CPM
CD163
MRC1
0 1 2 3 4 5 6 7 8 9 10 11 12
Identity
Percentile Expressed
25
50
75
100
Average Expression
2
1
0
−1

Supplementary Fig. 14| Marker genes for macrophage and DC cell clusters. Differentially expressed marker genes used for macrophage (a) and DC cells (b) were displayed. DC cells, dendritic cells.

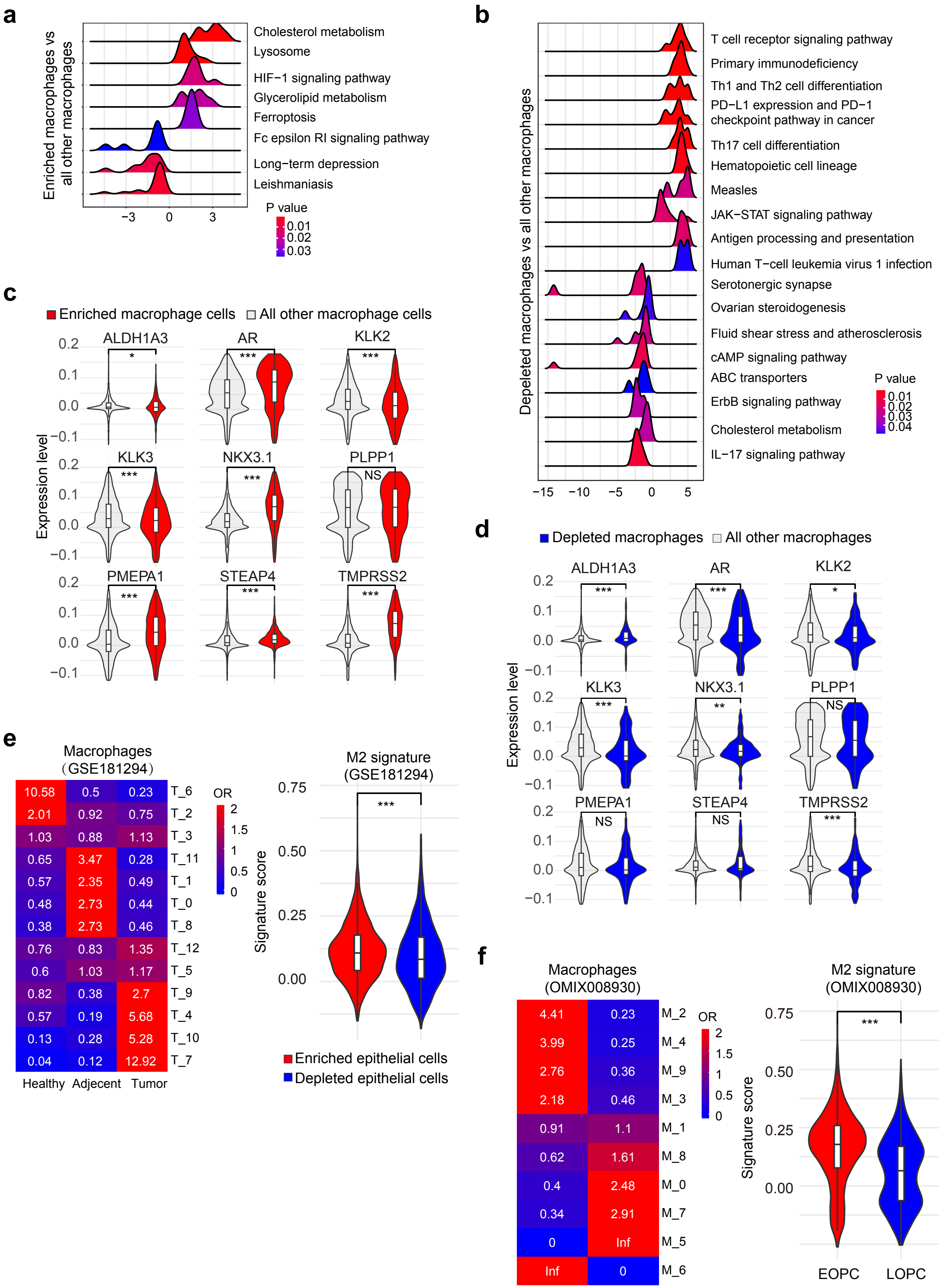

a
Enriched macrophages vs all other macrophages
Cholesterol metabolism
Lysosome
HIF−1 signaling pathway
Glycerolipid metabolism
Ferroptosis
Fc epsilon RI signaling pathway
Long−term depression
Leishmaniasis
−3
0
3
P value
0.01
0.02
0.03
b
Depeleted macrophages vs all other macrophages
T cell receptor signaling pathway
Primary immunodeficiency
Th1 and Th2 cell differentiation
PD−L1 expression and PD−1 checkpoint pathway in cancer
Th17 cell differentiation
Hematopoietic cell lineage
Measles
JAK−STAT signaling pathway
Antigen processing and presentation
Human T−cell leukemia virus 1 infection
Serotonergic synapse
Ovarian steroidogenesis
Fluid shear stress and atherosclerosis
cAMP signaling pathway
ABC transporters
ErbB signaling pathway
Cholesterol metabolism
IL−17 signaling pathway
P value
0.01
0.02
0.03
0.04
−15
−10
−5
0
5
c
Enriched macrophage cells
All other macrophage cells
Expression level
ALDH1A3 *
AR ***
KLK2 ***
KLK3 ***
NKX3.1 ***
PLPP1 NS
PMEPA1 ***
STEAP4 ***
TMPRSS2 ***
0.2
0.1
0.0
−0.1
d
Depleted macrophages
All other macrophages
Expression level
ALDH1A3 ***
AR ***
KLK2 *
KLK3 ***
NKX3.1 **
PLPP1 NS
PMEPA1 NS
STEAP4 NS
TMPRSS2 ***
e
Macrophages (GSE181294)
OR
2
1.5
1
0.5
0
T_6 10.58 0.5 0.23
T_2 2.01 0.92 0.75
T_3 1.03 0.88 1.13
T_11 0.65 3.47 0.28
T_1 0.57 2.35 0.49
T_0 0.48 2.73 0.44
T_8 0.38 2.73 0.46
T_12 0.76 0.83 1.35
T_5 0.6 1.03 1.17
T_9 0.82 0.38 2.7
T_4 0.57 0.19 5.68
T_10 0.13 0.28 5.28
T_7 0.04 0.12 12.92
Healthy
Adjecent
Tumor
M2 signature (GSE181294)
Signature score
0.75
0.50
0.25
0.00
***
Enriched epithelial cells
Depleted epithelial cells
f
Macrophages (OMIX008930)
M_2 4.41 0.23
M_4 3.99 0.25
M_9 2.76 0.36
M_3 2.18 0.46
M_1 0.91 1.1
M_8 0.62 1.61
M_0 0.4 2.48
M_7 0.34 2.91
M_5 0 Inf
M_6 Inf 0
OR
2
1.5
1
0.5
0
M2 signature (OMIX008930)
Signature score
0.75
0.50
0.25
0.00
***
EOPC
LOPC

Supplementary Fig. 15| Characteristics of the enriched and depleted clusters of macrophages in PTM samples. a, GSEA of enriched macrophage clusters in PTM samples (7,104 cells from 4 samples). **b,** GSEA of depleted macrophage clusters in PTM samples (7,104 cells from 4 samples). Pathway enrichment significance was determined using GSEA, with a nominal $P < 0.05$ and false discovery rate (FDR) < 0.25 considered statistically significant. **c,** Expression of androgen response genes in enriched macrophage clusters. Violin plots depict the kernel density of single-cell expression values. Median, 25th percentile, and 75th percentile are shown in the box plot; whiskers extending to 1.5 X IQR. Enriched Macrophages cells, 1,107 cells from 14 samples; all other Macrophage cells, 5,997 cells from 14 samples. **d,** Expression of androgen response genes in depleted macrophage clusters. Depleted Macrophages cells, 266 cells from 14 samples; all other Macrophage cells, 6,838 cells from 14 samples. **e,** Signature for M2 Macrophages is enriched in tumor areas but not healthy prostate or noncancerous adjacent areas. 8,062 macrophage cells from GSE181294 dataset (1) were used for analysis. **f,** M2 Macrophages are enriched in early onset prostate cancer (EOPC) but not late onset prostate cancer (LOPC). 3,287 cells from OMIX008930 dataset (2) were used for analysis. Wilcoxon test with Benjamini-Hochberg correction for P values. *, ** and *** denoted $P < 0.05$, $P < 0.01$ and $P < 0.001$, respectively.

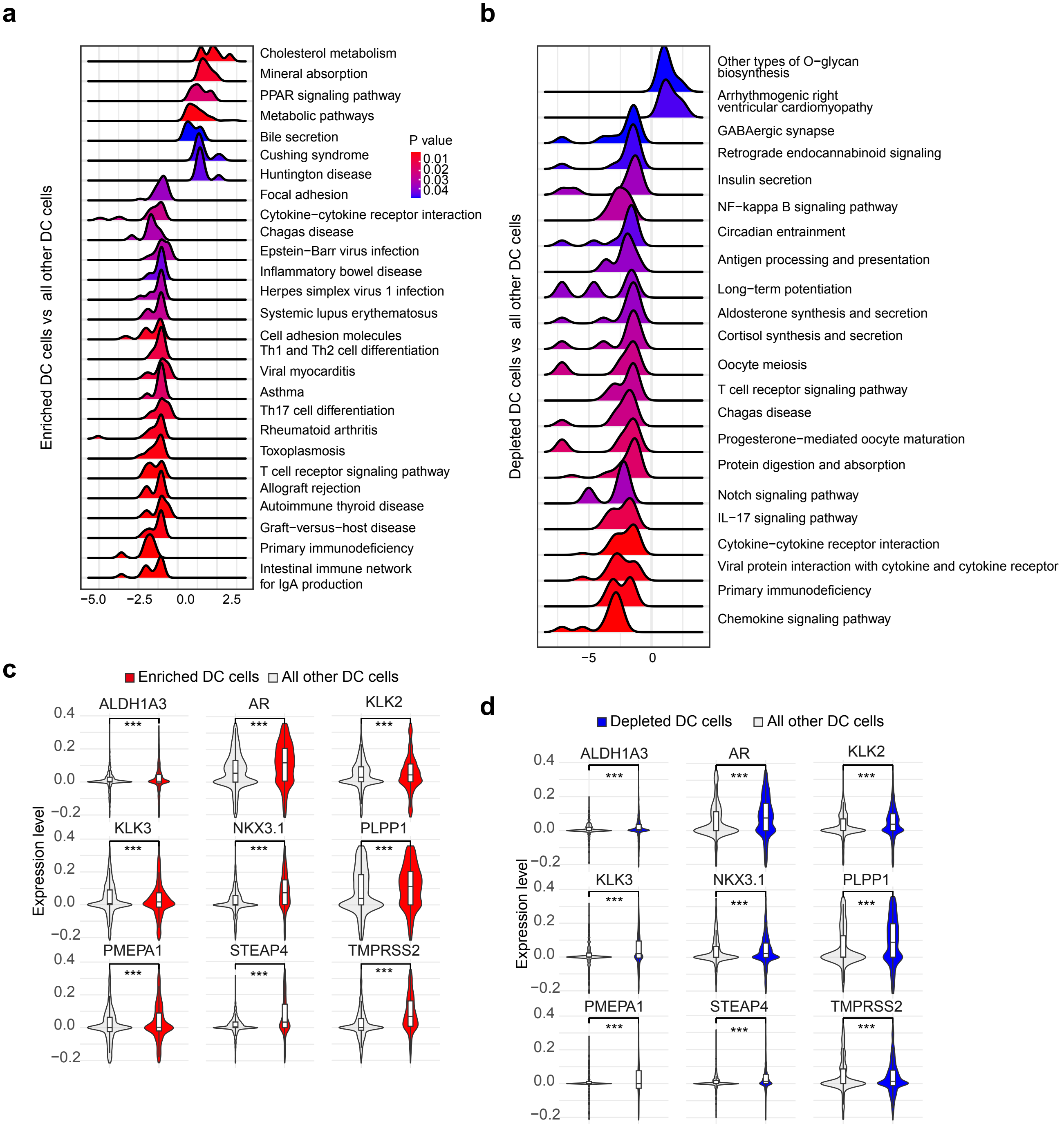

a
Cholesterol metabolism
Mineral absorption
PPAR signaling pathway
Metabolic pathways
Bile secretion
Cushing syndrome
Huntington disease
Focal adhesion
Cytokine−cytokine receptor interaction
Chagas disease
Epstein−Barr virus infection
Inflammatory bowel disease
Herpes simplex virus 1 infection
Systemic lupus erythematosus
Cell adhesion molecules
Th1 and Th2 cell differentiation
Viral myocarditis
Asthma
Th17 cell differentiation
Rheumatoid arthritis
Toxoplasmosis
T cell receptor signaling pathway
Allograft rejection
Autoimmune thyroid disease
Graft−versus−host disease
Primary immunodeficiency
Intestinal immune network for IgA production
P value
0.01
0.02
0.03
0.04
Enriched DC cells vs all other DC cells
−5.0
−2.5
0.0
2.5
b
Other types of O−glycan biosynthesis
Arrhythmogenic right ventricular cardiomyopathy
GABAergic synapse
Retrograde endocannabinoid signaling
Insulin secretion
NF−kappa B signaling pathway
Circadian entrainment
Antigen processing and presentation
Long−term potentiation
Aldosterone synthesis and secretion
Cortisol synthesis and secretion
Oocyte meiosis
T cell receptor signaling pathway
Chagas disease
Progesterone−mediated oocyte maturation
Protein digestion and absorption
Notch signaling pathway
IL−17 signaling pathway
Cytokine−cytokine receptor interaction
Viral protein interaction with cytokine and cytokine receptor
Primary immunodeficiency
Chemokine signaling pathway
Depleted DC cells vs all other DC cells
−5
0
c
Enriched DC cells
All other DC cells
ALDH1A3
AR
KLK2
KLK3
NKX3.1
PLPP1
PMEPA1
STEAP4
TMPRSS2
***
Expression level
0.4
0.2
0.0
−0.2
d
Depleted DC cells
All other DC cells
ALDH1A3
AR
KLK2
KLK3
NKX3.1
PLPP1
PMEPA1
STEAP4
TMPRSS2
***
Expression level
0.4
0.2
0.0
−0.2

Supplementary Fig. 16| Characteristics of the enriched and depleted clusters of DC cells in PTM samples. a, GSEA of enriched DC cell clusters in PTM samples (4,379 DC cells from 4 samples). **b,** GSEA of depleted DC cell clusters in PTM samples (4,379 DC cells from 4 samples). Statistical significance of pathway enrichment was determined using the GSEA method, with a nominal $P < 0.05$ and a false discovery rate (FDR) < 0.25 considered statistically significant. **c,** Expression of androgen response genes in enriched DC cell clusters. Violin plots depict the kernel density of single-cell expression values. Median, 25th percentile, and 75th percentile are shown in the box plot; whiskers extending to 1.5 X IQR. Enriched DC cells, 1,269 cells from 14 samples; All other DC cells, 3,110 cells from 14 samples. **d,** Expression of androgen response genes in depleted DC cell clusters. Depleted DC cells, 565 cells from 14 samples; All other DC cells, 3,814 cells from 14 samples. Wilcoxon test with Benjamini-Hochberg correction for P values. *, ** and *** denoted $P < 0.05$, $P < 0.01$ and $P < 0.001$, respectively.

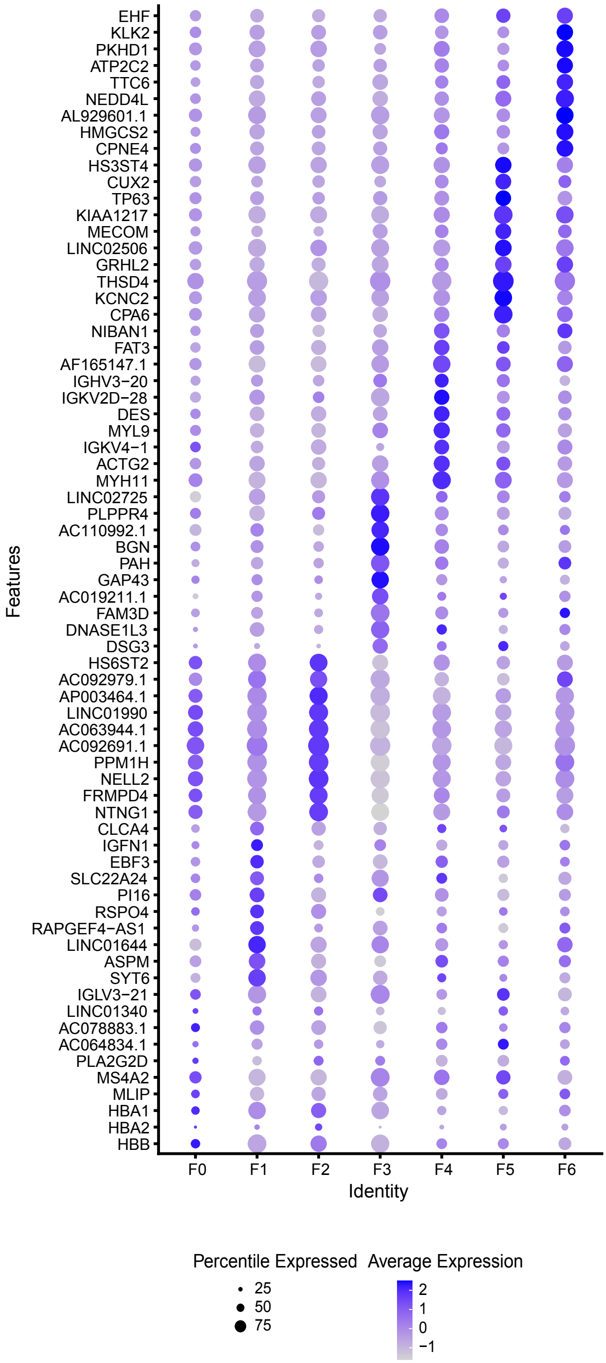
EHF
KLK2
PKHD1
ATP2C2
TTC6
NEDD4L
AL929601.1
HMGCS2
CPNE4
HS3ST4
CUX2
TP63
KIAA1217
MECOM
LINC02506
GRHL2
THSD4
KCNC2
CPA6
NIBAN1
FAT3
AF165147.1
IGHV3−20
IGKV2D−28
DES
MYL9
IGKV4−1
ACTG2
MYH11
LINC02725
PLPPR4
AC110992.1
BGN
PAH
GAP43
AC019211.1
FAM3D
DNASE1L3
DSG3
HS6ST2
AC092979.1
AP003464.1
LINC01990
AC063944.1
AC092691.1
PPM1H
NELL2
FRMPD4
NTNG1
CLCA4
IGFN1
EBF3
SLC22A24
PI16
RSPO4
RAPGEF4−AS1
LINC01644
ASPM
SYT6
IGLV3−21
LINC01340
AC078883.1
AC064834.1
PLA2G2D
MS4A2
MLIP
HBA1
HBA2
HBB
Features
F0
F1
F2
F3
F4
F5
F6
Identity
Percentile Expressed
25
50
75
Average Expression
2
1
0
−1

Supplementary Fig. 17| Differentially expressed marker genes used for unsupervised clustering of fibroblast cells.

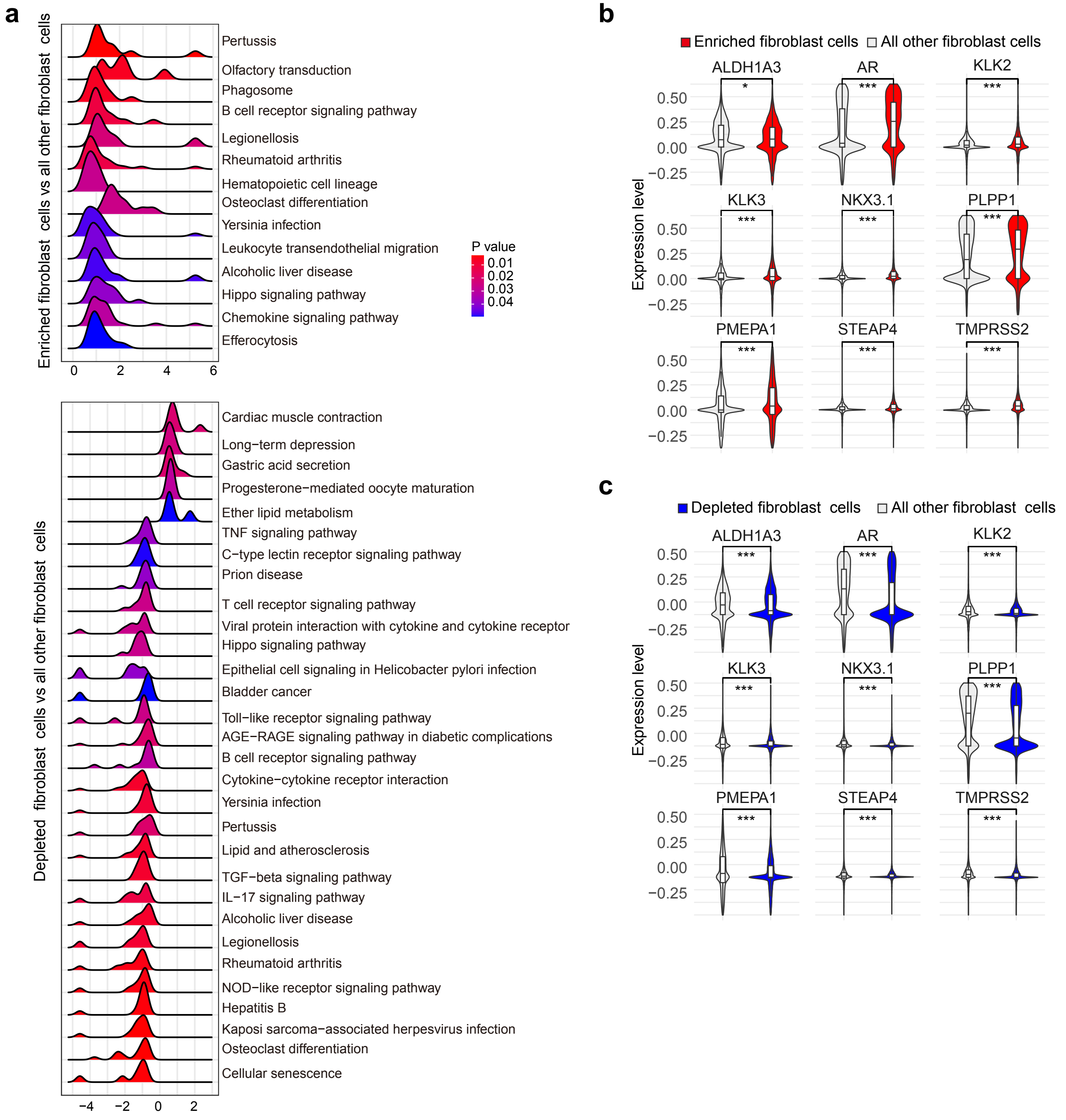
a
Enriched fibroblast cells vs all other fibroblast cells
Pertussis
Olfactory transduction
Phagosome
B cell receptor signaling pathway
Legionellosis
Rheumatoid arthritis
Hematopoietic cell lineage
Osteoclast differentiation
Yersinia infection
Leukocyte transendothelial migration
Alcoholic liver disease
Hippo signaling pathway
Chemokine signaling pathway
Efferocytosis
0
2
4
6
P value
0.01
0.02
0.03
0.04
Depleted fibroblast cells vs all other fibroblast cells
Cardiac muscle contraction
Long−term depression
Gastric acid secretion
Progesterone−mediated oocyte maturation
Ether lipid metabolism
TNF signaling pathway
C−type lectin receptor signaling pathway
Prion disease
T cell receptor signaling pathway
Viral protein interaction with cytokine and cytokine receptor
Hippo signaling pathway
Epithelial cell signaling in Helicobacter pylori infection
Bladder cancer
Toll−like receptor signaling pathway
AGE−RAGE signaling pathway in diabetic complications
B cell receptor signaling pathway
Cytokine−cytokine receptor interaction
Yersinia infection
Pertussis
Lipid and atherosclerosis
TGF−beta signaling pathway
IL−17 signaling pathway
Alcoholic liver disease
Legionellosis
Rheumatoid arthritis
NOD−like receptor signaling pathway
Hepatitis B
Kaposi sarcoma−associated herpesvirus infection
Osteoclast differentiation
Cellular senescence
−4
−2
0
2
b
Enriched fibroblast cells
All other fibroblast cells
Expression level
ALDH1A3
AR
KLK2
KLK3
NKX3.1
PLPP1
PMEPA1
STEAP4
TMPRSS2
*
***
0.50
0.25
0.00
−0.25
c
Depleted fibroblast cells
All other fibroblast cells
Expression level
ALDH1A3
AR
KLK2
KLK3
NKX3.1
PLPP1
PMEPA1
STEAP4
TMPRSS2
***
0.50
0.25
0.00
−0.25

Supplementary Fig. 18| Characteristics of fibroblast cells in three cohorts. a, GSEA of the enriched and depleted fibroblast cell clusters in PTM samples (n = 4). Statistical significance of pathway enrichment was determined using the GSEA method, with a nominal $P < 0.05$ and a false discovery rate (FDR) < 0.25 considered statistically significant. **b and c,** Expression of androgen response genes in the enriched or depleted fibroblast cell clusters from PTM samples (n = 4). Violin plots depict the kernel density of single-cell expression values. Median, 25th percentile, and 75th percentile are shown in the box plot; whiskers extending to 1.5 X IQR. Wilcoxon test with Benjamini-Hochberg for P value correction. *, ** and *** denoted $P < 0.05$, $P < 0.01$ and $P < 0.001$, respectively.

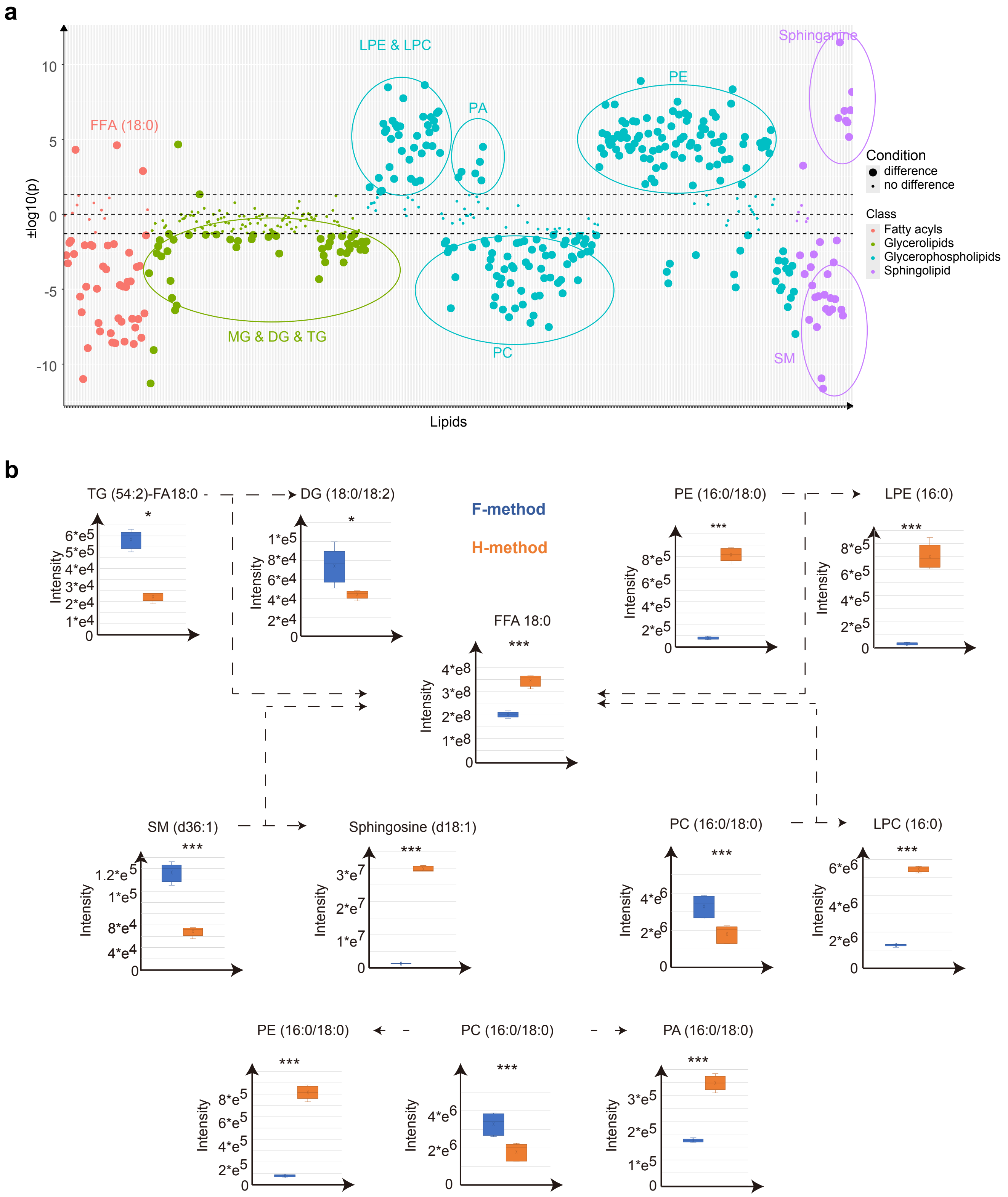

a
±log10(p)
Lipids
FFA (18:0)
LPE & LPC
PA
PE
Sphinganine
MG & DG & TG
PC
SM
Condition
difference
no difference
Class
Fatty acyls
Glycerolipids
Glycerophospholipids
Sphingolipid
b
F-method
H-method
TG (54:2)-FA18:0
DG (18:0/18:2)
PE (16:0/18:0)
LPE (16:0)
FFA 18:0
SM (d36:1)
Sphingosine (d18:1)
PC (16:0/18:0)
LPC (16:0)
PA (16:0/18:0)
Intensity

Supplementary Fig. 19| Development of mxFRIZNGRND method. a, Manhattan plot for the comparison of metabolites with F-extraction and H-extraction. Y-axis represents the statistical test ± Log (P value), if the metabolite is up-adjusted in the latter, a positive value is assigned, if the metabolite is down-adjusted, a negative value is assigned (n = 6). **b,** Comparison of metabolites detected by F-extraction and H-extraction (n = 6). Wilcoxon signed-rank test. For box plot, median, 25th percentile, and 75th percentile are shown; whiskers extend to the maximum and minimum values. *, $P < 0.05$; **, $P < 0.01$; ***, $P < 0.001$. TG, triglycerides; DAG, diacylglycerol; PE, phosphatidylethanolamine; LPE, lysophosphatidylethanolamine; FFA, free fatty acid; SM, sphingomyelin; PC, phosphatidylcholine; LPC, lysophosphatidylcholine; PE, phosphatidylserine; PA, phosphatidic acid.

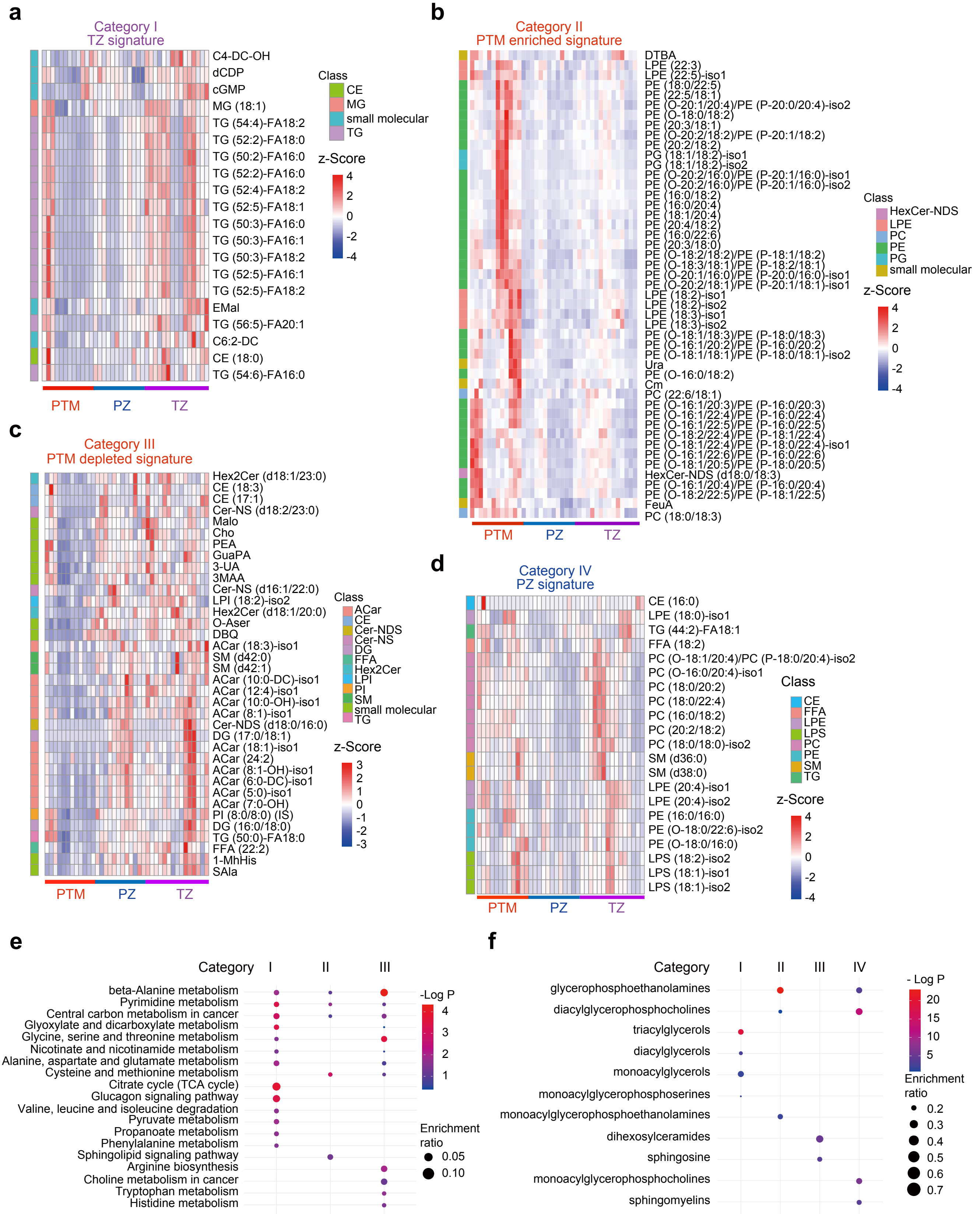
a
Category I
TZ signature
C4-DC-OH
dCDP
cGMP
MG (18:1)
TG (54:4)-FA18:2
TG (52:2)-FA18:0
TG (50:2)-FA16:0
TG (52:2)-FA16:0
TG (52:4)-FA18:2
TG (52:5)-FA18:1
TG (50:3)-FA16:0
TG (50:3)-FA16:1
TG (50:3)-FA18:2
TG (52:5)-FA16:1
TG (52:5)-FA18:2
EMal
TG (56:5)-FA20:1
C6:2-DC
CE (18:0)
TG (54:6)-FA16:0
PTM
PZ
TZ
Class
CE
MG
small molecular
TG
z-Score
4
2
0
-2
-4
b
Category II
PTM enriched signature
DTBA
LPE (22:3)
LPE (22:5)-iso1
PE (18:0/22:5)
PE (22:5/18:1)
PE (O-20:1/20:4)/PE (P-20:0/20:4)-iso2
PE (O-18:0/18:2)
PE (20:3/18:1)
PE (O-20:2/18:2)/PE (P-20:1/18:2)
PE (20:2/18:2)
PG (18:1/18:2)-iso1
PG (18:1/18:2)-iso2
PE (O-20:2/16:0)/PE (P-20:1/16:0)-iso1
PE (O-20:2/16:0)/PE (P-20:1/16:0)-iso2
PE (16:0/18:2)
PE (16:0/20:4)
PE (18:1/20:4)
PE (20:4/18:2)
PE (16:0/22:6)
PE (20:3/18:0)
PE (O-18:2/18:2)/PE (P-18:1/18:2)
PE (O-18:3/18:1)/PE (P-18:2/18:1)
PE (O-20:1/16:0)/PE (P-20:0/16:0)-iso1
PE (O-20:2/18:1)/PE (P-20:1/18:1)-iso1
LPE (18:2)-iso1
LPE (18:2)-iso2
LPE (18:3)-iso1
LPE (18:3)-iso2
PE (O-18:1/18:3)/PE (P-18:0/18:3)
PE (O-16:1/20:2)/PE (P-16:0/20:2)
PE (O-18:1/18:1)/PE (P-18:0/18:1)-iso2
Ura
PE (O-16:0/18:2)
Cm
PC (22:6/18:1)
PE (O-16:1/20:3)/PE (P-16:0/20:3)
PE (O-16:1/22:4)/PE (P-16:0/22:4)
PE (O-16:1/22:5)/PE (P-16:0/22:5)
PE (O-18:2/22:4)/PE (P-18:1/22:4)
PE (O-18:1/22:4)/PE (P-18:0/22:4)-iso1
PE (O-16:1/22:6)/PE (P-16:0/22:6)
PE (O-18:1/20:5)/PE (P-18:0/20:5)
HexCer-NDS (d18:0/18:3)
PE (O-16:1/20:4)/PE (P-16:0/20:4)
PE (O-18:2/22:5)/PE (P-18:1/22:5)
FeuA
PC (18:0/18:3)
PTM
PZ
TZ
Class
HexCer-NDS
LPE
PC
PE
PG
small molecular
z-Score
4
2
0
-2
-4
c
Category III
PTM depleted signature
Hex2Cer (d18:1/23:0)
CE (18:3)
CE (17:1)
Cer-NS (d18:2/23:0)
Malo
Cho
PEA
GuaPA
3-UA
3MAA
Cer-NS (d16:1/22:0)
LPI (18:2)-iso2
Hex2Cer (d18:1/20:0)
O-Aser
DBQ
ACar (18:3)-iso1
SM (d42:0)
SM (d42:1)
ACar (10:0-DC)-iso1
ACar (12:4)-iso1
ACar (10:0-OH)-iso1
ACar (8:1)-iso1
Cer-NDS (d18:0/16:0)
DG (17:0/18:1)
ACar (18:1)-iso1
ACar (24:2)
ACar (8:1-OH)-iso1
ACar (6:0-DC)-iso1
ACar (5:0)-iso1
ACar (7:0-OH)
PI (8:0/8:0) (IS)
DG (16:0/18:0)
TG (50:0)-FA18:0
FFA (22:2)
1-MhHis
SAla
PTM
PZ
TZ
Class
ACar
CE
Cer-NDS
Cer-NS
DG
FFA
Hex2Cer
LPI
PI
SM
small molecular
TG
z-Score
3
2
1
0
-1
-2
-3
d
Category IV
PZ signature
CE (16:0)
LPE (18:0)-iso1
TG (44:2)-FA18:1
FFA (18:2)
PC (O-18:1/20:4)/PC (P-18:0/20:4)-iso2
PC (O-16:0/20:4)-iso1
PC (18:0/20:2)
PC (18:0/22:4)
PC (16:0/18:2)
PC (20:2/18:2)
PC (18:0/18:0)-iso2
SM (d36:0)
SM (d38:0)
LPE (20:4)-iso1
LPE (20:4)-iso2
PE (16:0/16:0)
PE (O-18:0/22:6)-iso2
PE (O-18:0/16:0)
LPS (18:2)-iso2
LPS (18:1)-iso1
LPS (18:1)-iso2
PTM
PZ
TZ
Class
CE
FFA
LPE
LPS
PC
PE
SM
TG
z-Score
4
2
0
-2
-4
e
Category
I
II
III
beta-Alanine metabolism
Pyrimidine metabolism
Central carbon metabolism in cancer
Glyoxylate and dicarboxylate metabolism
Glycine, serine and threonine metabolism
Nicotinate and nicotinamide metabolism
Alanine, aspartate and glutamate metabolism
Cysteine and methionine metabolism
Citrate cycle (TCA cycle)
Glucagon signaling pathway
Valine, leucine and isoleucine degradation
Pyruvate metabolism
Propanoate metabolism
Phenylalanine metabolism
Sphingolipid signaling pathway
Arginine biosynthesis
Choline metabolism in cancer
Tryptophan metabolism
Histidine metabolism
-Log P
4
3
2
1
Enrichment ratio
0.05
0.10
f
Category
I
II
III
IV
glycerophosphoethanolamines
diacylglycerophosphocholines
triacylglycerols
diacylglycerols
monoacylglycerols
monoacylglycerophosphoserines
monoacylglycerophosphoethanolamines
dihexosylceramides
sphingosine
monoacylglycerophosphocholines
sphingomyelins
- Log P
20
15
10
5
Enrichment ratio
0.2
0.3
0.4
0.5
0.6
0.7

Supplementary Fig. 20| Metabolites for four signatures. a, Metabolites enriched in TZ samples (n = 6) (Category I as indicated in Fig. 7v). **b,** Metabolites enriched in PTM samples (n = 4) (Category II as indicated in Fig. 7v). **c,** Metabolites depleted in PTM samples (n = 4) (Category III as indicated in Fig. 7v). **d,** Metabolites depleted in PZ samples (n = 4) (Category IV as indicated in Fig. 7v). **e and f,** Metabolic pathways enriched in the metabolic signatures. Each sample was analyzed with three technical replicates.

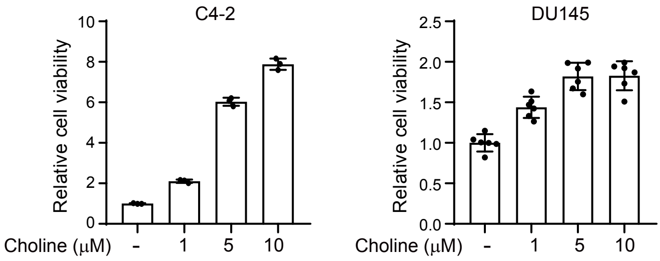
C4-2
Relative cell viability
10
8
6
4
2
0
Choline (μM)
-
1
5
10
DU145
Relative cell viability
2.5
2.0
1.5
1.0
0.5
0.0
Choline (μM)
-
1
5
10

Supplementary Fig. 21| Effect of choline in C4-2 and DU145 cells. Results were shown as mean ± SD; n = 3 and n = 6 independent replicates for C4-2 and DU145, respectively. Source data are provided as a Source Data file.

TIC plot of lipidome LC-MS method1 (C18)

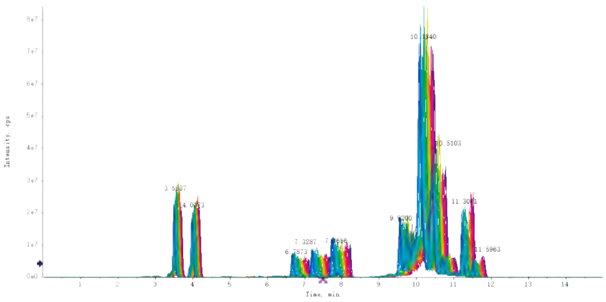

TIC plot of lipidome LC-MS method2 (HILIC)

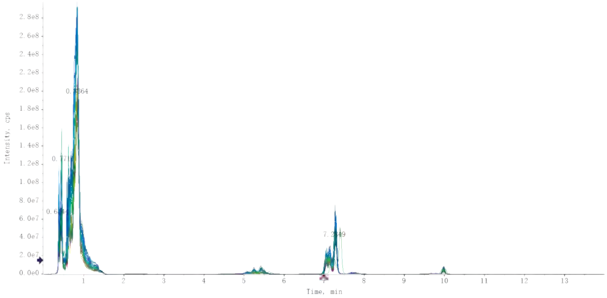

TIC plot of lipidome LC-MS method3 (EC C18)

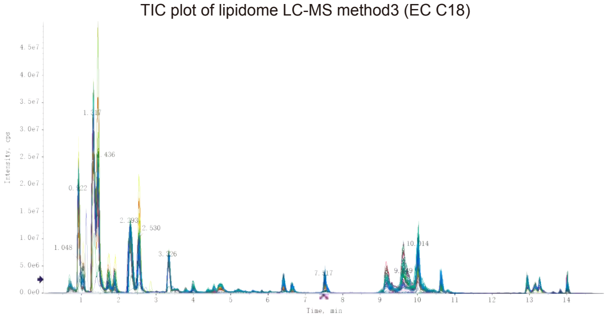

TIC plot of metabolome LC-MS method1 (negative ion model)

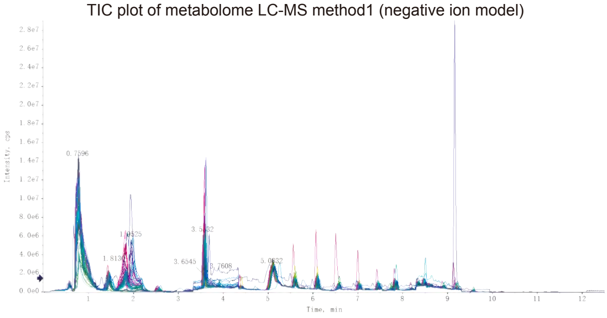

TIC plot of metabolome LC-MS method2 (positive ion model)

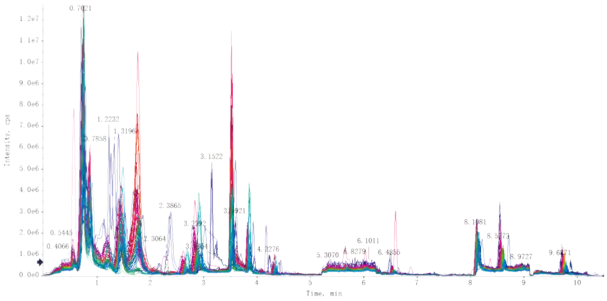

Supplementary Fig. 22| Representative total ion chromatograms (TICs) for LC-MS methods.

Supplementary Table 1| Baseline characteristics for patients in Fig. 1a.

Localized prostate cancer cohort (N = 235)	Zone 1: PZ N = 142 (60.4%)	Zone 2: TZ N = 59 (25.1%)	Zone 3: PZ+TZ N = 34 (14.5%)	Significance (P value)
Age (mean ± sd)	69.01 (6.36)	68.51 (7.64)	68.85 (5.42)	0.9[1]
Prostate Volume (mean ± sd)	40.10 (24.85)	38.17 (18.02)	34.23 (15.10)	0.5[1]
PSA (mean ± sd)	10.89 (7.81)	13.47 (11.43)	13.88 (8.43)	0.014[1]
Gleason score: n (%)				0.4[2]
3+3	23 (16%)	14 (24%)	5 (15%)	
3+4	47 (33%)	26 (44%)	14 (41%)	
4+3	47 (33%)	11 (19%)	10 (29%)	
≥4+4	25 (18%)	8 (14%)	5 (15%)	
T stage: n (%)				0.004[3]
T1	15 (11%)	5 (8.5%)	1 (2.9%)	
T2a	73 (51%)	17 (29%)	11 (32%)	
T2b	7 (4.9%)	4 (6.8%)	3 (8.8%)	
T2c	14 (9.9%)	5 (8.5%)	8 (24%)	
T3b	33 (23%)	28 (47%)	11 (32%)	

[1] Two side Kruskal-Wallis rank sum test; [2] Two side Pearson's Chi-squared test; [3] Two side Fisher's Exact Test for Count Data with simulated p-value (based on 2000 replicates).

Supplementary Table 2| **Baseline characteristics for patients in Fig. 1b.**

Neoadjuvant cohort (N = 198)	Zone 1: PZ<br>N = 87 (43.9%)	Zone 2: TZ<br>N = 31 (15.7%)	Zone 3: PZ+TZ<br>N = 80 (40.4%)	Significance (P value)
Age (mean ± sd)	68.92 (6.94)	71.23 (4.20)	68.61 (6.09)	0.2[1]
Prostate Volume (mean ± sd)	41.18 (16.75)	45.52 (21.15)	42.36 (20.18)	0.6[1]
PSA (mean ± sd)	73.51 (119.11)	48.90 (35.36)	70.39 (65.91)	0.3[1]
Gleason score: n (%)				0.9[2]
≤ 3+4	3 (3.5%)	3 (9.7%)	5 (6.3%)	
4+3	16 (19%)	7 (23%)	15 (19%)	
4+4	43 (50%)	14 (45%)	41 (51%)	
≥ 4+5	24 (28%)	7 (23%)	19 (24%)	
T stage: n (%)				0.12[3]
≤ T3a	29 (33%)	18 (58%)	29 (36%)	
T3b	45 (52%)	8 (26%)	39 (49%)	
T4	13 (15%)	5 (16%)	12 (15%)	

[1] Two side Kruskal-Wallis rank sum test; [2] Two side Pearson's Chi-squared test; [3] Two side Fisher's Exact Test for Count Data with simulated p-value (based on 2000 replicates).

Metabolites abbreviations:

Full name	Abbreviation
Acylcarnitine	ACar
Triglyceride	TG
Phosphatidylcholine	PC
Phosphatidylethanolamine	PE
Sphingomyelin	SM
Diacylglycerol	DG
Monoacylglycerol	MG
Lysophosphatidylcholine	LPC
Lysophosphatidylethanolamine	LPE
Sphingosine	Sph
Phosphatidylglycerol	PG
Hexosyl 2 ceramide	Hex2Cer
Lysophosphatidic acid	LPA
4-hydroxy-phenylglycine	4-HPGly
3-Methoxytyramine	3-MeTyr
Dihydrothymine	DHT
L-Asparagine	Asn
O-Phosphoserine	O-PS
Acetyllysine	AcLys
Methionine	Met
S-adenosylhomocysteine	SAH
trans-ferulic acid	FeuA
Methylthioadenosine	MTAdo
cyclic-3',5'- GMP	cGMP
Nicotinate	NicoA
Glucosamine	GlcN
Picolinic acid	PicoA
O-Acetylserine	O-Aser
Quinic acid	QA
1-methyluric acid	1-MUA
N,N-Dimethyltryptamine	NN-Dtry
Serylserine	Ser-Ser
L-Thiaproline	Thiap
N-Formyl-D-phenylalanine	NFDPh
N-Acetylmannosamine	ManNAc
1-pyrroline-5-carboxylic acid	1-PyCA
Acylcarnitine-FFA24:2	C24:2
Acylcarnitine-FFA4 with double carboxyl groups	C4-DC-OH

Supplementary References

1. Hirz, T., Mei, S., Sarkar, H., Kfoury, Y., Wu, S., Verhoeven, B.M., Subtelny, A.O., Zlatev, D.V., Wszolek, M.W., Salari, K. et al. (2023) Dissecting the immune suppressive human prostate tumor microenvironment via integrated single-cell and spatial transcriptomic analyses. *Nature communications*, 14, 663.

2. Cheng, Y., Liu, B., Xin, J., Wu, X., Li, W., Shang, J., Wu, J., Zhang, Z., Xu, B., Du, M. *et al.* (2025) Single-cell and spatial RNA sequencing identify divergent microenvironments and progression signatures in early- versus late-onset prostate cancer. *Nature aging*, 5, 909-928.
