# Supplementary material for: Analysis of the transcriptomic and metabolomic landscape of prostate cancer with different anatomical origins using snFLARE-seq and mxFRIZNGRND
Source: Nat Commun. 2026 Feb 7;17:2461. doi: 10.1038/s41467-026-69347-7 (PMC12992566; doi:10.1038/s41467-026-69347-7)
Supplement: Supplementary file 2 — Description of Additional Supplementary Information [file 41467_2026_69347_MOESM2_ESM.pdf]

Description of Additional Supplementary Information

Supplementary Data 1| Distribution of immune cells for multicolor staining.

Supplementary Data 2| The list of detected metabolites with their abbreviations, full names, and class.

Supplementary Data 3| The standard list of lipidome.

Supplementary Data 4| Comprehensive profiling of metabolite abundances across different tumor sources. (QC CV <30%)

Supplementary Data 5| Comprehensive profiling of lipid concentration across different tumor sources. (QC CV <30%)

Supplementary Data 6| MS spectral data for metabolites.
