# Supplementary material for: Analysis of the transcriptomic and metabolomic landscape of prostate cancer with different anatomical origins using snFLARE-seq and mxFRIZNGRND
Source: Nat Commun. 2026 Feb 7;17:2461. doi: 10.1038/s41467-026-69347-7 (PMC12992566; doi:10.1038/s41467-026-69347-7)
Supplement: Supplementary file 4 — Reporting Summary [file 41467_2026_69347_MOESM4_ESM.pdf]

nature portfolio

Corresponding author(s): Zhenfei Li

Last updated by author(s): Jan 28, 2026

Reporting Summary

Nature Portfolio wishes to improve the reproducibility of the work that we publish. This form provides structure for consistency and transparency in reporting. For further information on Nature Portfolio policies, see our Editorial Policies and the Editorial Policy Checklist.

Please do not complete any field with "not applicable" or n/a. Refer to the help text for what text to use if an item is not relevant to your study.
For final submission: please carefully check your responses for accuracy; you will not be able to make changes later.

Statistics

For all statistical analyses, confirm that the following items are present in the figure legend, table legend, main text, or Methods section.

n/a	Confirmed	
☐	☒	The exact sample size (*n*) for each experimental group/condition, given as a discrete number and unit of measurement
☐	☒	A statement on whether measurements were taken from distinct samples or whether the same sample was measured repeatedly
☐	☒	The statistical test(s) used AND whether they are one- or two-sided<br>*Only common tests should be described solely by name; describe more complex techniques in the Methods section.*
☐	☒	A description of all covariates tested
☐	☒	A description of any assumptions or corrections, such as tests of normality and adjustment for multiple comparisons
☐	☒	A full description of the statistical parameters including central tendency (e.g. means) or other basic estimates (e.g. regression coefficient) AND variation (e.g. standard deviation) or associated estimates of uncertainty (e.g. confidence intervals)
☐	☒	For null hypothesis testing, the test statistic (e.g. *F*, *t*, *r*) with confidence intervals, effect sizes, degrees of freedom and *P* value noted<br>*Give P values as exact values whenever suitable.*
☒	☐	For Bayesian analysis, information on the choice of priors and Markov chain Monte Carlo settings
☒	☐	For hierarchical and complex designs, identification of the appropriate level for tests and full reporting of outcomes
☐	☒	Estimates of effect sizes (e.g. Cohen's *d*, Pearson's *r*), indicating how they were calculated

Our web collection on statistics for biologists contains articles on many of the points above.

Software and code

Policy information about availability of computer code

Data collection: List in the Method

Data analysis: List in the Method

For manuscripts utilizing custom algorithms or software that are central to the research but not yet described in published literature, software must be made available to editors and reviewers. We strongly encourage code deposition in a community repository (e.g. GitHub). See the Nature Portfolio guidelines for submitting code & software for further information.

Data

Policy information about availability of data

All manuscripts must include a data availability statement. This statement should provide the following information, where applicable:
- Accession codes, unique identifiers, or web links for publicly available datasets
- A description of any restrictions on data availability
- For clinical datasets or third party data, please ensure that the statement adheres to our policy

The metabolomic data generated in this study have been deposited in the OMIX database under accession code OMIX013309 (https://ngdc.cncb.ac.cn/omix/release/OMIX013309) and OMIX013310 (https://ngdc.cncb.ac.cn/omix/release/OMIX013310). The transcriptomic data generated in this study have been deposited in the Gene Expression Omnibus (GEO) database at the National Center for Biotechnology Information (NCBI) under accession number GSE317733 (https://www.ncbi.nlm.nih.gov/geo/query/acc.cgi?acc=GSE317733). These data are also already publicly available in the National Omics Data Encyclopedia (https:// www.biosino.org/node; accession OEP 00005630). Datasets derived from TCGA are available at the cBioPortal website (www.cbioportal.org/). CPGEA sequencing data are available in the Biological Project Library (PRJCA001124) (https://ngdc.cncb.ac.cn/bioproject/browse/PRJCA001124) (41). GRCh38 human reference genome was download from 10X genomics (https://support.10xgenomics.com/single-cell-gene expression/software/downloads). The publicly available single-cell RNA

sequencing data used in this study and related signatures for M2 macrophage, Antigen_Presenting, Exhaustion T cells, and Treg are accessible from the NCBI Gene Expression Omnibus database GSE181294 (https://www.ncbi.nlm.nih.gov/geo/query/acc.cgi?acc=GSE181294) (39). The prostate cancer publicly available data

in this study are available in the OMIX database under accession code OMIX008930 (https://ngdc.cncb.ac.cn/omix/release/OMIX008930) (40). The publicly available bulk RNA-seq for prostate cancer (GSE21034) are from the NCBI Gene Expression Omnibus database (https://www.ncbi.nlm.nih.gov/geo/query/acc.cgi?acc=GSE21034) (98). Spectra data for metabolites is shown in Supplementary Data 6. Source Data are provided with this paper.

Research involving human participants, their data, or biological material

Policy information about studies with human participants or human data. See also policy information about sex, gender (identity/presentation), and sexual orientation and race, ethnicity and racism.

Reporting on sex and gender: Prostate cancer research limited in male

Reporting on race, ethnicity, or other socially relevant groupings: Chinese patients were the only

Population characteristics: Included in Supplementary table 1-2

Recruitment: Patients with localized prosate cancer were selected and assigend into two groups acco

Ethics oversight: Patient-derived tissues were collected in adherence to the guidelines establishec

Note that full information on the approval of the study protocol must also be provided in the manuscript.

Field-specific reporting

Please select the one below that is the best fit for your research. If you are not sure, read the appropriate sections before making your selection.

- [x] Life sciences
- [ ] Behavioural & social sciences
- [ ] Ecological, evolutionary & environmental sciences

For a reference copy of the document with all sections, see nature.com/documents/nr-reporting-summary-flat.pdf

Life sciences study design

All studies must disclose on these points even when the disclosure is negative.

Sample size: To investigate the clinical responses of prostate cancer from different origins to pro

Data exclusions: Patients with immediate postoperative anti-androgen therapy (n = 3) or no postoper

Replication: Experiments were performed at least three

Randomization: N

Blinding: Investigators for sequencing and metabolites detection were blind to the clinical

Reporting for specific materials, systems and methods

We require information from authors about some types of materials, experimental systems and methods used in many studies. Here, indicate whether each material, system or method listed is relevant to your study. If you are not sure if a list item applies to your research, read the appropriate section before selecting a response.

Materials & experimental systems

n/a	Involved in the study	
●	☐	Antibodies
☐	●	Eukaryotic cell lines
●	☐	Palaeontology and archaeology
●	☐	Animals and other organisms
☐	●	Clinical data
●	☐	Dual use research of concern
●	☐	Plants

Methods

n/a	Involved in the study	
●	☐	ChIP-seq
●	☐	Flow cytometry
●	☐	MRI-based neuroimaging

Eukaryotic cell lines

Policy information about cell lines and Sex and Gender in Research

Cell line source(s): LNCaP and C4-2 were purchased from the American Type Culture Collection (ATCC,

Authentication: Authentication of cell lines was confirmed by Hybribio (Guangzhou,

Mycoplasma contamination: mycoplasma-free status was verified using specific primers: 5'- GGGAGCAAA

Commonly misidentified lines (See ICLAC register): *Name any commonly misidentified cell lines used in the study and provide a rationale for their use.*

Clinical data

Policy information about clinical studies

All manuscripts should comply with the ICMJE guidelines for publication of clinical research and a completed CONSORT checklist must be included with all submissions.

Clinical trial registration: N

Study protocol: Note where the full trial protocol can be accessed OR if not available, explain

Data collection Patient-derived tissues were collected in adherence to the guidelines established

Outcomes BC

Plants

Seed stocks n

Novel plant genotypes na

Authentication na

This checklist template is licensed under a Creative Commons Attribution 4.0 International License, which permits use, sharing, adaptation, distribution and reproduction in any medium or format, as long as you give appropriate credit to the original author(s) and the source, provide a link to the Creative Commons license, and indicate if changes were made. The images or other third party material in this article are included in the article's Creative Commons license, unless indicated otherwise in a credit line to the material. If material is not included in the article's Creative Commons license and your intended use is not permitted by statutory regulation or exceeds the permitted use, you will need to obtain permission directly from the copyright holder. To view a copy of this license, visit http://creativecommons.org/licenses/bv/4.0/
